# Two‐Dimensional Atomically Thin Piezoelectric Nanosheets for Efficient Pyroptosis‐Dominated Sonopiezoelectric Cancer Therapy

**DOI:** 10.1002/advs.202405741

**Published:** 2024-09-09

**Authors:** Ruxi Deng, Chunrong Ren, Xinran Song, Wuyang Wei, Hai Wang, Quanyu Nie, Ying Liu, Pan Li, Li Ding, Meiqi Chang, Yu Chen, Yang Zhou

**Affiliations:** ^1^ Department of Ultrasound The Third People's Hospital of Chengdu Affiliated Hospital of Southwest Jiaotong University Chengdu Sichuan 610031 P. R. China; ^2^ Department of Gastroenterology The Third People's Hospital of Chengdu Affiliated Hospital of Southwest Jiaotong University Chengdu Sichuan 610031 P. R. China; ^3^ Materdicine Lab School of Life Sciences Shanghai University Shanghai 200444 P. R. China; ^4^ Department of Ultrasound Chongqing Key Laboratory of Ultrasound Molecular Imaging the Second Affiliated Hospital of Chongqing Medical University Chongqing 400010 P. R. China; ^5^ Department of Medical Ultrasound National Clinical Research Center of Interventional Medicine Shanghai Tenth People's Hospital Tongji University Cancer Center School of Medicine Tongji University Shanghai 200072 P. R. China; ^6^ Laboratory Center Shanghai Municipal Hospital of Traditional Chinese Medicine Shanghai University of Traditional Chinese Medicine Shanghai 200071 P. R. China; ^7^ Shanghai Institute of Materdicine Shanghai 200051 P. R. China

**Keywords:** Bi_2_O_2_(OH)(NO_3_) nanosheets, pyroptosis, sonopiezocatalytic therapy, ultrasound, ultrathin

## Abstract

Sonopiezocatalytic therapy is an emerging therapeutic strategy that utilizes ultrasound irradiation to activate piezoelectric materials, inducing polarization and energy band bending to facilitate the generation of reactive oxygen species (ROS). However, the efficient generation of ROS is hindered by the long distance of charge migration from the bulk to the material surface. Herein, atomically thin Bi_2_O_2_(OH)(NO_3_) (AT‐BON) nanosheets are rationally engineered through disrupting the weaker hydrogen bonds within the [OH] and [NO_3_] layer in the bulk material. The ultrathin structure of AT‐BON piezocatalytic nanosheets shortens the migration distance of carriers, expands the specific surface area, and accelerates the charge transfer efficiency, showcasing a natural advantage in ROS generation. Importantly, the non‐centrosymmetric polar crystal structure grants the nanosheets the ability to separate electron‐hole pairs. Under ultrasonic mechanical stress, Bi_2_O_2_(OH)(NO_3_) nanosheets with the remarkable piezoelectric feature exhibit the desirable in vivo antineoplastic outcomes in both breast cancer model and liver cancer model. Especially, the AT‐BON‐induced ROS bursts lead to the activation of the Caspase‐1‐driven pyroptosis pathway. This study highlights the beneficial impact of bulk material thinning on enhancing ROS generation efficiency and anti‐cancer effects.

## Graphical abstract

This work reports on the engineering of atomically thin Bi_2_O_2_(OH)(NO_3_) nanosheets as ROS generator and pyroptosis inducer for sonopiezoelectric cancer therapy through shortening the migration distance of carriers, expanding the specific surface area, and accelerating the charge transfer efficiency.

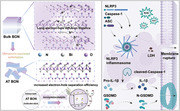

## 1 Introduction

Piezoelectric catalytic materials, when activated by mechanical energy, have the capability to generate electrons‐holes and produce reactive oxygen species (ROS).^[^ ^1^ ^]^ These ROS‐induced oxidative stress can modulate biological processes within cells, such as cell necrosis, apoptosis, pyroptosis and oxidation of lipids and proteins. Consequently, the accumulation of ROS at tumor sites can facilitate effective tumor therapy.^[^ ^2^ ^]^ Ultrasound (US), a common external stimulus, interacts with piezoelectric materials, inducing polarization and generating a built‐in electric field. This process leads to the continuous separation of electrons and holes, which are then attracted to opposite surfaces, ultimately resulting in the generation of ROS when reacting with surrounding molecules such as H_2_O and O_2_. Furthermore, in comparison to conventional photodynamic therapy, which suffers from limited penetration depth and lacks precision adjustment, sonopiezoelectric catalysis demonstrates superior tissue penetration and spatial controllability.^[^ ^3^ ^]^ These characteristics render sonopiezoelectric catalysis an attractive field for biomedical research.

Sonopiezoelectric nanomaterials, such as BaTiO_3_,^[^ ^4^ ^]^ Bi_2_MoO_6_,^[^ ^5^ ^]^ (K,Na)NbO_3_,^[^ ^6^ ^]^ and ZnO,^[^ ^7^ ^]^ have been rationally engineered for tumor therapy. Unlike many nanomedicines with diverse compositions and complex nanostructures, these piezoelectric nanomaterials offer a straightforward, well‐defined composition and structure, along with adjustable morphology and size.^[^ ^8^ ^]^ Moreover, the strong light‐matter interactions and high carrier mobilities render 2D materials highly suitable for applications in the field of nano‐biomedicine. According to the energy band theory of the piezoelectric catalytic mechanism, the piezoelectric material is polarized under US stimulation. The piezoelectric potential, generated by the piezoelectric effect, determines the energy levels of the valence band (VB) and conduction band (CB) of the material. This facilitates charge exchange at the material interface, enabling an efficient catalytic redox reaction. However, certain therapeutic piezoelectric catalysts may fail to achieve desired outcomes due to either an insufficiently high critical Gibbs free energy threshold from periodic mechanical forces to initiate redox reactions or weak reaction efficiency resulting in inadequate redox levels.^[^ ^9^ ^]^ Researchers have attempted to enhance the piezoelectric properties of these materials through methods such as introducing defects,^[^ ^10^ ^]^ doping with metal ions^[^ ^11^ ^]^ and constructing heterogeneous structures.^[^ ^12^ ^]^ However, the complexity of preparation and the mixing of structural components remain unavoidable challenges.

Bi_2_O_2_(OH)(NO_3_) (abbreviated as BON), an emerging photocatalyst, is composed of [Bi_2_O_2_(OH)]^+^layers interleaved with NO_3_^−^ along the z‐axis. This non‐centrosymmetric polar crystal structure naturally facilitates the separation of electron‐hole pairs.^[^ ^13^ ^]^ Nevertheless, the excessive thickness of the BON nanosheets impedes efficient charge migration from the bulk to the surface.^[^ ^14^ ^]^ Constructing a thin‐layer structure can significantly reduce the charge diffusion distance, accelerate the charge transfer rates, impede the charge recombination, and diminish the resistance of carriers reaching the active sites on the surface across each layer.^[^ ^15^ ^]^ In light of these advantages, BON with ultrathin structure holds high promise as a sonopiezoelectric material for the efficient generation of ROS.

**Scheme 1:**
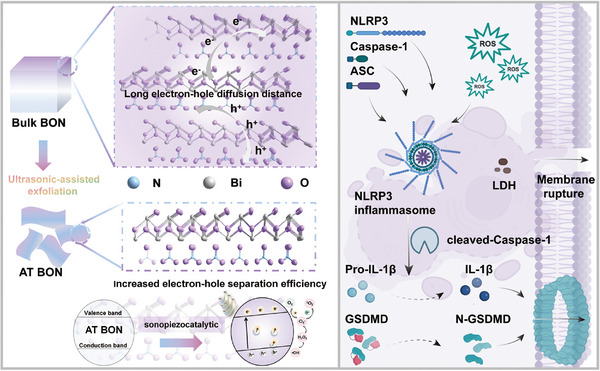
Schematic diagram of the 2D atomically thin Bi_2_O_2_(OH)(NO_3_) (AT‐BON) piezoelectric nanosheets for sonopiezocatalytic therapy. BON nanosheets with atomically thin feature shorted carrier diffusion distance and accelerated the separation of electron‐hole pairs, amplifying the ROS generation and activating the NLRP3‐Caspase‐1‐GSDMD pyroptosis pathway.

Herein, we fabricated 2D atomically thin BON (referred to as AT‐BON) nanosheets with a non‐centrosymmetric polar crystal structure by disrupting the weaker hydrogen bonds within the [OH] and [NO_3_] layer.^[^ ^15^ ^]^ The stimulation of US polarizes AT‐BON, enabling efficient sonopiezoelectric catalysis through the displacement of charge centers.^[^ ^16^ ^]^ The AT‐BON nanosheets with remarkable piezoelectric property exhibit the desirable in vivo antineoplastic outcomes in both breast cancer model and liver cancer model under ultrasonic mechanical stress. Especially, the gene sequencing analysis revealed that the anti‐tumor mechanism of AT‐BON is intricately associated with pyroptosis, a form of programmed cell death mediated by gasdermin. This process entails cell swelling, plasma membrane pore formation, and membrane rupture (**Scheme** **1**). Western blot analysis and enzyme‐linked immunosorbent assay (ELISA) kit assessment confirmed that AT‐BON induced pyroptosis via the classical pathway involving caspase‐1 activation by the NLRP3 inflammasome. This study highlights the importance of thinning piezoelectric materials in amplifying the production of ROS and inducing the cell pyroptosis process.

## 2 Results and Discussion

### 2.1 Synthesis and Characterization of AT‐BON NSs

**Figure 1:**
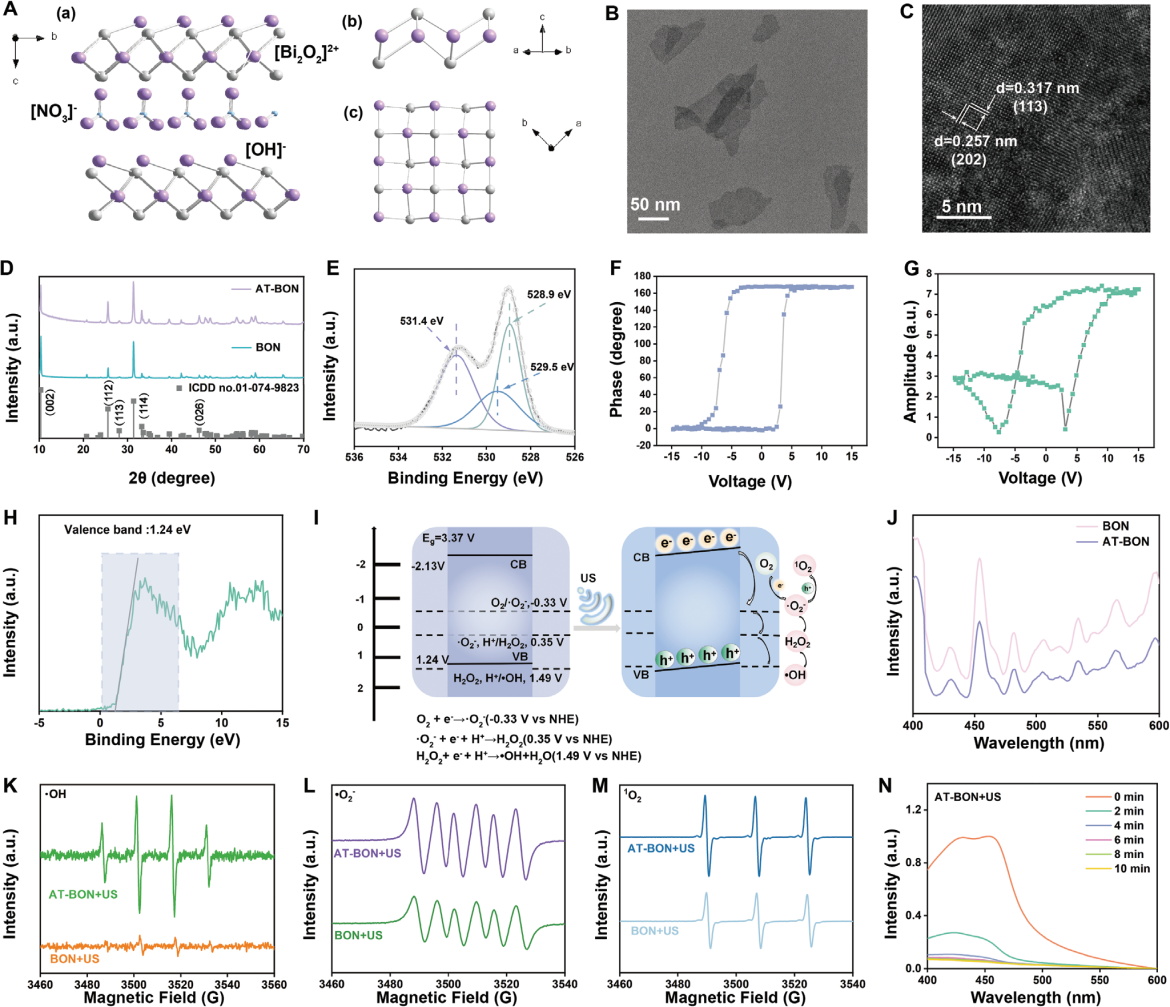
Synthesis and characterization of AT‐BON. A) Schematic crystal structure of AT‐BON: a) 3D projection; b,c) [Bi_2_O_2_]^2+^ layers along the [110] and [001] direction, respectively. B) TEM and C) HRSTEM images of AT‐BON. D) X‐ray diffraction pattern of AT‐BON and BON. E) XPS spectra of O 1s of AT‐BON. F) Piezoresponsive phase curve and G) amplitude curve of AT‐BON. H) Valence XPS spectra of AT‐BON. I) Piezocatalytic mechanism of AT‐BON under ultrasonic excitation and corresponding energy band diagrams. J) Fluorescence spectra of BON and AT‐BON. ESR spectra of K) •OH and L) •O_2_^−^ trapped by DMPO and M) ^1^O_2_ trapped by TEMP. N) Degradation of DPBF under AT‐BON + US treatment with different US durations.

AT‐BON nanosheet was synthesized by a facile ultrasound‐assisted top‐down method (Figure S1, Supporting Information). The bulk Bi_2_O_2_(OH)(NO_3_) was initially synthesized using a straightforward hydrothermal method. Considering that the [OH] layer and the [NO_3_] layer are linked by comparatively weak hydrogen bonds due to the limited overlap of H 1s orbitals with the electronic states of [NO_3_] groups, the aqueous dispersion of the resulting product underwent a 4‐h ultrasonic peeling treatment to disrupt the hydrogen bonds. Finally, AT‐BON NSs were obtained by a process of differential centrifugation. The crystal structure indicates that AT‐BON has a non‐centrosymmetric structure consisting of a [Bi_2_O_2_]^2+^ layer and interspersed OH^−^ and (NO_3_)^−^ groups (**Figure** **1**A). Distinguished from the bulk BON with block morphology (Figure S2, Supporting Information), transmission electron microscopy (TEM) image reveals a uniform nanosheet topology of AT‐BON (Figure 1B). The thickness and 3D morphology were assessed using atomic force microscopy (AFM). AT‐BON exhibits an irregular layered structure in the planar image (Figure S3A, Supporting Information). Both the 3D image and the corresponding height profile demonstrate that the height of AT‐BON is about 1.2 nm, further confirming its atomic layer‐thin structure (Figure S3B, Supporting Information).

Dynamic light scattering (DLS) measurements reveal that the hydrodynamic size and surface potential of AT‐BON is 189 nm and −26.7 ± 0.83 mV, respectively (Figure S4, Supporting Information). High‐resolution scanning TEM (HRSTEM) image reveals lattice spacings of 0.257 and 0.317 nm, respectively, corresponding to the (202) and (113) planes of AT‐BON (Figure 1C). The successful synthesis of AT‐BON was further confirmed by the characteristic signals and uniform distribution of Bi, N, and O as detected in the energy‐dispersive X‐ray spectroscopy (Figure S5, Supporting Information). Afterward, the crystal structures of AT‐BON and BON were demonstrated by X‐ray diffraction (XRD) pattern (Figure 1D). The diffraction peaks at 10.316°, 25.546°, 28.1117°, 31.378°, and 46.290°, respectively, correspond to (002), (112), (113), (114), and (026) planes, which suggests that the crystal structure is not significantly altered by ultrasonic stripping. Furthermore, Raman spectra of BON and AT‐BON were recorded to further validate the structure change (Figure S6, Supporting Information). Bi‐O or Bi‐OH stretching modes (peaks at 123, 160, 200, 375, 427, 470 cm^−1^) and the vibration of NO_3_^−^ (705 cm^−1^) are consistent in BON and AT‐BON, indicating that the structure of AT‐BON remained unchanged after stripping. X‐ray photoelectron spectroscopy (XPS) measurements also reveal the chemical composition of the surface of AT‐BON. The survey spectrum of AT‐BON confirms the presence of Bi, N, and O (Figure S7A, Supporting Information). For the high‐resolution spectrum of Bi 4f (Figure S7B, Supporting Information), two sub‐peaks at 164.7 and 159.4 eV are assigned to Bi 4f_5/2_ and Bi 4f_7/2_, respectively.^[^ ^15^ ^]^ The O 1s spectrum of AT‐BON can be decomposed into three peaks at 531.9, 529.5, and 528.9 eV (Figure 1E), which are assigned to surface‐adsorbed oxygen, oxygen vacancy and lattice oxygen, respectively.^[^ ^17^ ^]^ In contrast, peaks at 532.2 and 530.0 eV corresponding to surface‐adsorbed oxygen and lattice oxygen have been detected in the bulk BON (Figure S8, Supporting Information), confirming the important role of material thinning in the generation of oxygen vacancies.

The piezoelectric properties of AT‐BON were evaluated by piezoelectric force microscopy (PFM). Typical butterfly shaped phase‐voltage and amplitude‐voltage hysteresis loops demonstrate the piezoelectric characteristics (Figure 1F,G). The optical properties of AT‐BON were analyzed through UV absorption spectrum in the range of 200–800 nm, where a broadband absorption at 200–400 nm emerges because of the relatively high band gap energy and the excitation of electrons from the valence band to the conduction band (Figure S9A, Supporting Information). The band gap value of AT‐BON was recorded as 3.37 eV using the derivative Tauc plot (Figure S9B, Supporting Information). Besides, the position of valence band was elucidated by the XPS valence band spectra, and the corresponding value is 1.24 V (Figure 1H). Figure 1I is a schematic diagram of the intrinsic energy bands and the tilted energy bands under US stimulation of AT‐BON NSs. Alterations in local dipole moments induce piezoelectric polarization, resulting in the generation of a built‐in piezoelectric field within the AT‐BON. The generated electrons react with O_2_ to form •O_2_^−^, and the intermediate •O_2_^−^ reacts with the electrons to produce H_2_O_2_, which can then undergo further reduction to •OH.^[^ ^18^ ^]^ In addition, •O_2_^−^ can also react with the holes to produce ^1^O_2_.^[^ ^19^ ^]^ The fluorescence spectra indicated that the emission peaks of AT‐BON were weaker than those of BON, which implied that material thinning process improves the carrier separation efficiency (Figure 1J).

### 2.2 ROS Generation Capacity of AT‐BON NSs

Considering that ultra‐thin lamellar structure of AT‐BON NS provides a charge‐rich surface and facilitates the effective separation of electron‐hole pairs, the ROS generation capacity has been systematically assessed to verify the sonopiezoelectric catalytic effect. Methylene blue (MB) fading experiments have been initially executed through comparing the normalized absorption intensities at 664 nm (Figure S10A–C, Supporting Information). As envisioned, the control group exhibited no significant changes after 10 min of US irradiation. Following the establishment of adsorption‐desorption equilibrium between AT‐BON NSs and MB molecules, the group subjected to US excitation experienced a notable reduction in absorption over time compared to the group without US exposure. The degradation rate of MB by AT‐BON NSs reached 48% after 10 min of US irradiation (Figure S10D, Supporting Information). Significantly, AT‐BON significantly improved the degradation rate of MB compared to bulk materials, confirming the effective generation of ROS (Figure S11, Supporting Information). Electron spin resonance (ESR) techniques were employed to definitively identify the specific ROS generated through charge separation under US irradiation. 5,5‐dimethyl‐1‐pyrroline‐N‐oxide (DMPO) served as the trapping agent for •OH and •O_2_^−^, whereas 2,2,6,6‐tetramethylpiperidine (TEMP) was utilized to capture ^1^O_2_. Figure 1K–M indicates the presence of characteristic signal peaks corresponding to •OH, •O_2_^−^ and ^1^O_2_ in the AT‐BON + US group, which are much stronger than those of BON + US group, providing evidence on generating these three types of ROS through interactions with H_2_O and O_2_ under US excitation. Next, the 1,3‐diphenylisobenzofuran (DPBF) probe was employed for additional validation of ^1^O_2_ production (Figure 1N, Figure S12A,B, Supporting Information). Consistent with previous findings, the absorption peak of DPBF at 445 nm in the AT‐BON + US group gradually declined with prolonging of US irradiation duration. The degradation rate of DPBF reached 90% within 4 min of US irradiation (Figure S12C, Supporting Information). Furthermore, it was further verified that AT‐BON produced more ^1^O_2_ than BON after US irradiation for the same period of time (Figure S13, Supporting Information). The experimental findings above demonstrated that AT‐BON NSs featured a strong piezoelectric response, enabling effective electron‐hole separation and the generation of abundant ROS.

### 2.3 In Vitro Sonopiezoelectric Performance

Given the remarkably high efficiency in ROS production by AT‐BON NSs, we selected the mouse breast cancer cell line 4T1 and the mouse liver cancer cell line Hepa1‐6 as representative cell lines for assessing anti‐tumor efficacy. We improved the dispersibility and biocompatibility of the nanosheets by applying a coating of polyvinyl pyrrolidone (PVP) to the surface of AT‐BON NSs, referred to as AT‐BON@P (Figure S14, Supporting Information). Prior to commencing in vitro treatment, we verified the cell endocytosis process. Through observation using bio‐transmission electron microscopy, it was confirmed that AT‐BON@P NSs effectively entered the 4T1 cells (Figure S15, Supporting Information). Furthermore, the time‐dependent internalization procedure has been verified through observing the co‐localization of Hepa1‐6 cells and rhodamine B‐labelled AT‐BON@P NSs (Figure S16, Supporting Information).

**Figure 2:**
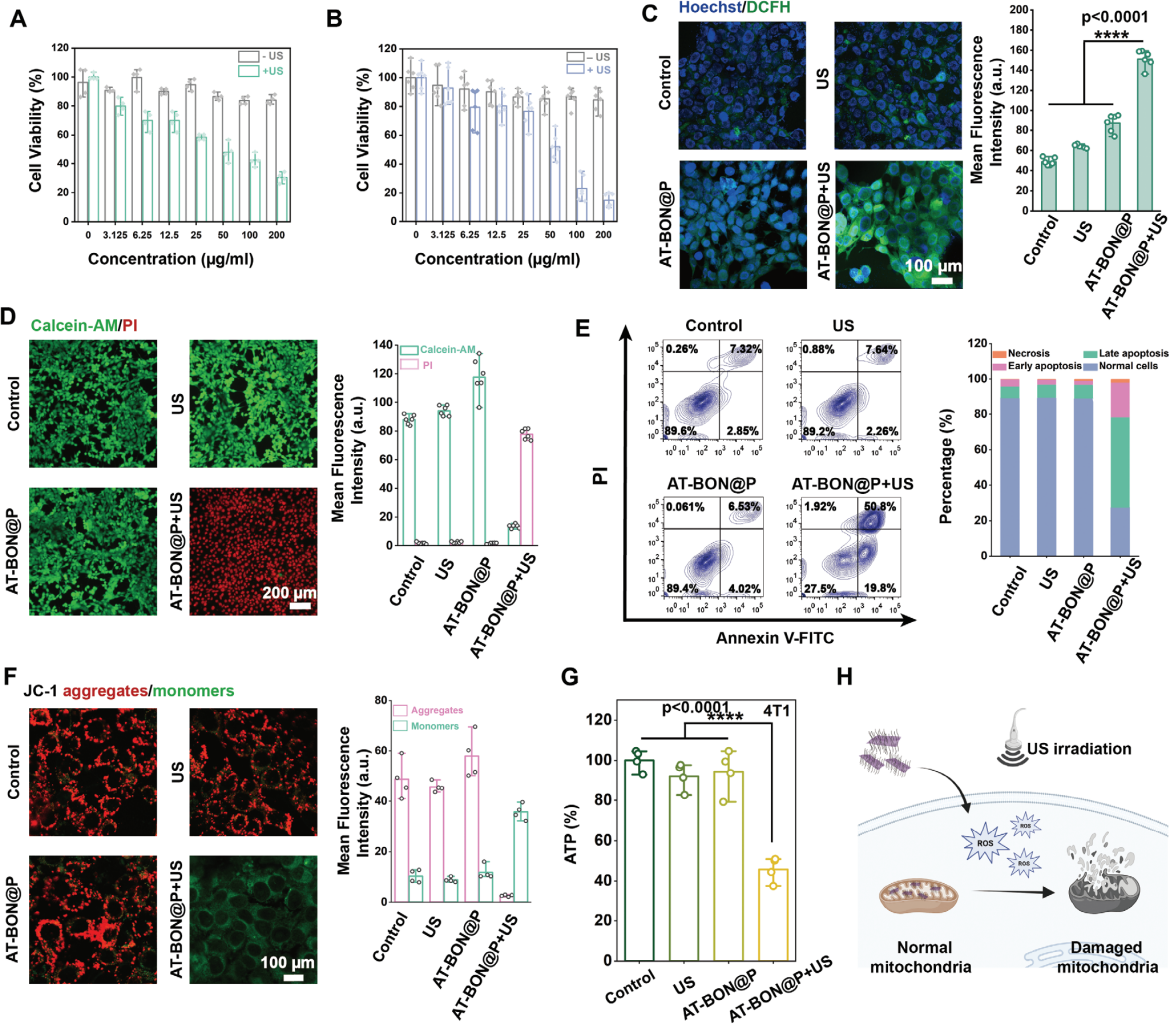
In vitro assessment. Cytotoxicity of AT‐BON@P against A) 4T1 and B) Hepa1‐6 cells with or without US treatment. C) Confocal images and the corresponding fluorescence semi‐quantitation data of 4T1 cells stained with DCFH‐DA after different treatments. D) Calcein AM/PI staining and the corresponding fluorescence semi‐quantitation data of 4T1 cells after different treatments. E) Flow cytometric analysis of 4T1 cells co‐stained with Annexin V‐FITC and PI after different treatments and the corresponding semi‐quantitation data. F) JC‐1 staining and the corresponding fluorescence semi‐quantitation of 4T1 cells after different treatments. G) The intracellular ATP levels of 4T1 cells after various treatments. H) Schematic representation of US‐assisted mitochondrial damage caused by sonopiezoelectric effect. Statistical significances were calculated via Student's *t*‐test. ****p < 0.0001.

Next, the in vitro cytotoxicity of nanosheets was evaluated using a typical cell counting kit 8 (CCK‐8) protocol (**Figure** **2**A,B). Both 4T1 and Hepa1‐6 cells demonstrated the sustained viability even after 24 h of co‐incubation, even at the concentrations of as high as 200 µg mL^−1^, indicating the favorable biosafety profile of AT‐BON@P NSs. However, after the addition of US irradiation, the cell viability of 4T1 and Hepa1‐6 decreased significantly, reaching 30.42% and 15%, respectively. This observation serves as preliminary validation of the antitumor effect of AT‐BON NS. Intracellular ROS levels were assessed using 2′,7′‐dichlorofluorescein diacetate (DCFH‐DA) as a probe. Consistent results were observed in both 4T1 (Figure 2C) and Hepa1‐6 (Figure S17, Supporting Information) cell lines. Minimal fluorescence was detected in cells treated with either AT‐BON NSs or US alone. However, upon exposure to US irradiation, AT‐BON@P NSs exhibited a significant increase in green fluorescence indicative of ROS production, thereby confirming the piezoelectric catalysis. Following confirmation of toxic ROS production, cell viability was assessed via dual staining with green fluorescent calcein acetoxymethyl ester (Calcein AM, indicating living cells) and red fluorescence using propidium iodide (PI, indicating dead cells). As expected, the substantial red fluorescence observed in the AT‐BON@P+US group demonstrates that the sonopiezoelectric catalytic effect led to extensive cell death (Figure 2D, Figure S18, Supporting Information). Typical flow apoptosis assays have been performed through membrane‐linked protein V‐FITC and PI staining (Figure 2E). In the AT‐BON@P+US group of 4T1 cells, there were 19.8% of early apoptotic cells and 50.8% of late apoptotic cells, while the Hepa1‐6 cells showed 43.1% of early apoptosis and 1.75% of late apoptosis (Figure S19, Supporting Information).

We subsequently employed the 5,5′,6,6′‐tetrachloro‐1,1′,3,3′‐tetraethylbenzimidazolo carbocyanine iodide (JC‐1) probe to assess the functional state of mitochondria, as the production of ROS typically leads to mitochondrial damage (Figure 2H).^[^ ^20^ ^]^ In AT‐BON@P NSs under US irradiation, the mitochondrial membrane potential displayed an abnormal state and exhibited green fluorescence due to loss or reduction in the AT‐BON@P + US groups (Figure 2F and Figure S20, Supporting Information). In contrast, the normal mitochondria of the remaining group emitted intense red fluorescence due to the aggregation of JC‐1 in the mitochondrial matrix. This observation was further confirmed by the quantitative fluorescence analysis. Intracellular adenosine triphosphate (ATP) levels are indicative of mitochondrial metabolic status. Thus, we investigated intracellular ATP levels in 4T1 and Hepa1‐6 cells following various treatments (Figure 2G and Figure S21, Supporting Information). As anticipated, ATP synthesis in both cancer cell lines experienced varying degrees of inhibition.

### 2.4 Therapeutic Mechanism

**Figure 3:**
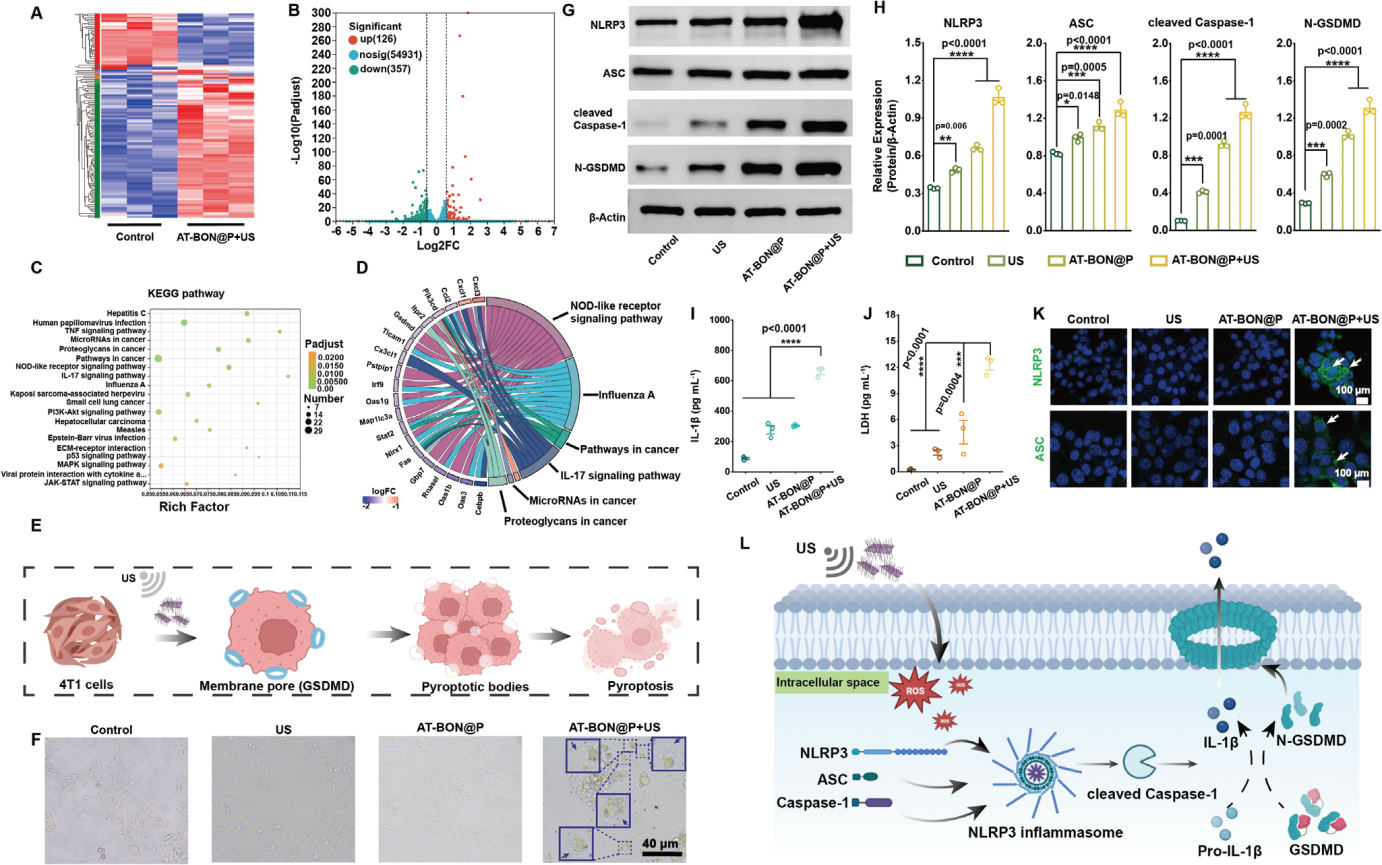
Therapeutic mechanism. A) Heat map diagram of differentially expressed mRNAs screened from control group and AT‐BON@P + US group (*n* = 3). B) Volcano plot of expression differences between AT‐BON@P + US and control groups. C) KEGG enrichment analysis showing the top 20 pathways in which differentially expressed genes. D) Enriched chord diagram of the KEGG pathways. E) Schematic diagram of the generation of plasma membrane pore. F) Morphology changes of 4T1 cells after different treatments. Blue boxes indicate pyroptotic cells. G) Western blot analysis of NLRP3, ASC, cleaved Caspase‐1, and N‐GSDMD in 4T1 cells after varied treatments. H) The corresponding quantitative analysis of NLRP3, ASC, cleaved Caspase‐1, and N‐GSDMD protein expressions based on western blotting results (*n* = 3). I) IL‐1β (*n* = 3) and J) LDH release (*n* = 3) after being treated with different groups. K) Representative immunofluorescence of NLRP3 and ASC in 4T1 cells. The white arrows indicate speck‐like aggregates of NLRP3 and ASC. L) Schematic representation of pyroptosis pathway induced by AT‐BON@P under US excitation. Statistical significances were calculated via Student's *t*‐test. *p < 0.1, **p < 0.01, ***p < 0.001, ****p < 0.0001.

For a more detailed comprehension of the biological mechanism underlying AT‐BON@P NSs‐mediated piezo‐catalyzed tumor therapy, RNA‐seq analysis of gene expression in 4T1 cancer cells has been carried out. The heat map suggests significant transcriptomic disparities between the treatment and control cohorts (**Figure** **3**A). a total of 483 differentially expressed genes (DEGs) were identified, with 126 up‐regulated genes (red) and 357 down‐regulated genes (blue) (Figure 3B). The results from the KEGG pathway enrichment analysis revealed that the differential genes between treatment group and control group were enriched in inflammation‐related signaling pathways, such as NOD‐like receptor signaling pathway, TNF signaling pathway, IL‐17 signaling pathway (Figure 3C). By plotting the string diagram of the KEGG pathway analysis alongside the specific regulatory genes corresponding to the major influencing pathways, it can be seen that the most significant regulatory genes can be attributed to the NOD‐like receptor signaling pathway (Figure 3D). Given the sequencing results presented above, we are compelled to associate the mechanism of AT‐BON@P action on 4T1 cells with pyroptosis, a crucial innate immune response distinct from apoptosis or necrosis, likely resulting in the release of substantial pro‐inflammatory factors.^[^ ^21^ ^]^

The classical pyroptotic cell pathway entails the initial recruitment and activation of Caspase‐1 protein,^[^ ^22^ ^]^ subsequent activation of inflammatory factors, and direct cleavage and activation of Gasdermin family proteins. These proteins form pores in the cell membrane, enabling the slow efflux of cell contents and ultimately resulting in pyroptosis.^[^ ^23^ ^]^ Our subsequent objective is to meticulously gather compelling evidence for AT‐BON@P NS‐induced pyroptosis. Morphologically, pyroptotic cells exhibit swelling and enlargement, along with numerous bubble‐like protrusions^[^ ^24^ ^]^ (Figure 3E). Biological electron microscopy observation revealed that the 4T1 cell membrane exhibited partial porosity following treatment with AT‐BON@P + US group, leading to the leakage of intracellular material through the pores and the generation of numerous pyroptotic bodies (Figure 3F). The representative pyroptotic balloon‐like cells demonstrated significant activation of the cellular pyroptotic pathway.

In order to further elucidate the mechanism of action, we employed a protein blotting assay to identify representative proteins associated with pyroptosis. The reduced expression of pro‐Caspase‐1 and elevated expressions of nucleotide‐binding domain and leucine‐rich repeat family pyrin domain containing 3 (NLRP3) inflammasome‐related proteins, apoptosis‐associated speckle‐like protein (ASC, an adaptor protein), and cleaved Caspase‐1 were observed in the AT‐BON@P +US group (Figure S22, Supporting Information, Figure 3G,H). Confocal immunofluorescence assays further confirmed the activation of NLRP3 and elevated ASC activity (Figure 3K). N‐GSDMD forms highly aggregated structures and binds to membrane lipids, resulting in the perforation of the cell membrane, leading to changes in cellular osmotic pressure and progressive swelling until the eventual rupture of the cell membrane and the release of cell contents, such as lactate dehydrogenase (LDH) and interleukin (IL)−1β.^[^ ^25^ ^]^ Elevated IL‐1β levels were observed in the AT‐BON@P+US group compared to the other groups, indicating the maturation and release of the IL‐1β, a key signal for pyroptosis (Figure 3I). LDH, a stable cytoplasmic enzyme found in all cells, is released upon damage to cell membranes and is a key indicator of cellular damage. The enzyme‐linked immunosorbent assay (ELISA) revealed elevated LDH levels in the AT‐BON@P+US group (Figure 3J). The above results confirm that AT‐BON@P+US induces cellular pyroptosis through the classical pathway of Caspase‐1 (Figure 3L). It can be reasonably inferred that Bi_2_O_2_(OH)(NO_3_) 2D piezoelectric nanosheets with ultrasound‐induced ROS generation ability can be used as a pyroptosis inducer through destroying the mitochondrial function of tumor cells and enhancing the intracellular accumulation of ROS.

### 2.5 In Vivo Sonopiezoelectric Therapy

**Figure 4:**
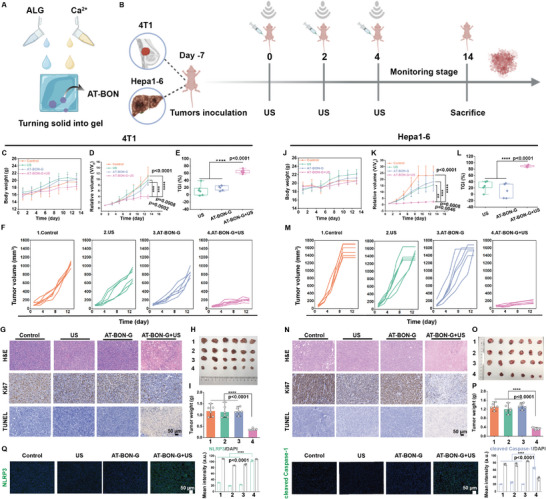
In vivo assessment and mechanism research. A) Schematic representation of the preparation process of AT‐BON‐G. B) Schematic illustration of the treatment strategy toward 4T1 and Hepa1‐6 tumor‐bearing mice. C) Time‐dependent body weight, D) Relative tumor volumes, E) tumor growth inhibition (TGI), and F) individual tumor‐growth curves of 4T1‐tumor‐bearing mice after various treatments (Control group; US group (1 MHz, 1.2 W cm^−2^, 50% duty cycle, and 5 min); AT‐BON‐G group; AT‐BON‐G + US group). The error bars are based on the SD (*n* = 6). I) Time‐dependent body weight, J) tumor volumes, L) TGI, and M) individual tumor‐growth curves of Hepa1‐6‐tumor‐bearing mice after various treatments (Control group; US group (1 MHz, 1.2 W cm^−2^, 50% duty cycle, and 5 min); AT‐BON‐G group; AT‐BON‐G + US group). The error bars are based on the SD (*n* = 6). On the eighth day, certain mouse tumors had grown to a size that exceeded or were on the verge of exceeding the volume considered ethical. Consequently, euthanasia protocols were implemented. G) 4T1 and N) Hepa1‐6 tumor slices stained by H&E, Ki67, and TUNEL with different treatments. Photos of the excised H) 4T1 and O) Hepa1‐6 tumors after indicated treatments. Weight of the dissected I) 4T1 and P) Hepa1‐6 tumors of the mice after treatment. Q) NLRP3 and cleaved Caspase‐1 staining images of 4T1‐tumor‐bearing mice and the corresponding fluorescence semi‐quantitative results in different treatment groups. Statistical significances were calculated via Student's *t*‐test. *p < 0.1, **p < 0.01, ***p < 0.001, ****p < 0.0001.

Building upon these results, we proceeded to assess the anticancer efficacy of AT‐BON NSs in vivo using 4T1 breast cancer and Hepa1‐6 hepatic carcinoma models. The conversion of solids into gels for tumor treatment has been reported.^[^ ^26^ ^]^ Consequently, we employed a similar strategy to convert AT‐BON into injectable AT‐BON‐hydrogel (AT‐BON‐G) for sonopiezoelectric therapy (**Figure** **4**A). The prepared AT‐BON‐G with favorable injectability can readily accumulate in tumors while inducing fewer side effects resulting from nanosheet penetration into blood and other tissues. 4T1‐ or Hepa1‐6‐tumor‐bearing mice were randomly assigned into the following four experimental groups: 1) control group; 2) US group; 3) AT‐BON‐G group; and 4) AT‐BON‐G + US group. US irradiation (1.0 MHz, 1.2 W cm^−2^, 50% duty cycle) was performed 1 h after intratumoral injection of AT‐BON‐G for 5 min on days 1, 3, 5, and 7, respectively (Figure 4B). The biosafety assessment results indicated that weight fluctuations during the treatment period were not significant in all groups (Figure 4C–J). Hematoxylin‐eosin (H&E) staining of major organs (heart, liver, spleen, lung and kidney) revealed no significant signs of organ toxicity associated with AT‐BON‐G (Figures S23 and S24, Supporting Information).

AT‐BON‐G + US group exhibited significant tumor suppression in both 4T1 breast cancer and Hepa1‐6 liver cancer models throughout a 14‐day observation and monitoring period (Figure 4D,K). At the end of day 14 treatment period, the AT‐BON‐G + US group exhibited a significant tumor growth inhibition (TGI) rate, with values of 64.5% for 4T1 and 89.5% for Hepa1‐6, surpassing those of the US group (12.67% for 4T1; 25.5% for Hepa1‐6) and the AT‐BON‐G group (19.5% for 4T1; 14.4% for Hepa1‐6) (Figure 4E,L). In addition, the survival of AT‐BON‐G + US group was significantly prolonged in the Hepa1‐6 liver cancer mouse model (Figure S25, Supporting Information). The excellent tumor suppression effect of AT‐BON‐G was further corroborated by photographic evidence (Figure 4H,O) and tumor weight measurements (Figure 4I,P).

Subsequently, histological examinations including H&E immunochemistry, Ki‐67 and terminal deoxynucleotidyl transferase (TdT)‐mediated dUTP nick‐end labeling (TUNEL) staining were implemented to offer a comprehensive interpretation of the antitumor observations. As shown in the Figure 4G,N, both 4T1 breast cancer and Hepa1‐6 liver cancer exhibited the most extensive cell damage and necrosis in the AT‐BON‐G + US group. The expression of the Ki67 protein, which serves as an indicator of cell proliferation, was significantly inhibited. Furthermore, the highest percentage of apoptotic cells in the AT‐BON‐G + US group has been confirmed through TUNEL staining. Subsequently, we conducted in vivo immunofluorescence assays to assess the expression of related proteins associated with pyroptosis. In the AT‐BON‐G + US group, NLRP3 inflammatory vesicles and Caspase‐1 were obviously activated. This activation could lead to the release of inflammatory factors, triggering an inflammatory response that ultimately leads to pyroptosis (Figure 4Q).

## 3 Conclusion

In summary, 2D atomically thin Bi_2_O_2_(OH)(NO_3_) piezoelectric nanosheets with accelerated charge transfer efficiency have been rationally designed and engineered for sonopiezocatalytic tumor therapy. Bi_2_O_2_(OH)(NO_3_) nanosheets exfoliated from the bulk material exhibited shortened migration distance and substantial generation of ROS. Transcriptome sequencing results and western blot analysis demonstrated the Caspase‐1/GSDMD‐dependent pyroptosis pathway induced by sonopiezocatalytic effect, ultimately leading to tumor cell death and achieving tumor suppression. Both in vitro cellular experiments and in vivo animal assessments (breast cancer model and liver cancer model) showcased remarkable tumor‐therapeutic outcomes. This study highlights the significant role of piezoelectric material thinning in enhancing the efficacy of ROS‐dominated tumor treatment.

## Conflict of Interest

The authors declare no conflict of interest.

## Author Contributions

R.D., C.R., and X.S. contributed equally to this work. M.C., Y.C., and Y.Z. designed and supervised the project and commented on it. R.D., C.R., W.W., H.W., and X.S. performed the experiments. R.D., Y.L., P.L., L.D., Q.N., and X.S. analyzed the data. R.D. wrote the experiment section. All authors participated in the manuscript preparation and contributed to the discussions during the project.

## Supporting information

Supporting Information

## Data Availability

The data that support the findings of this study are available from the corresponding author upon reasonable request.
