# Supplementary material for: Two‐Dimensional Atomically Thin Piezoelectric Nanosheets for Efficient Pyroptosis‐Dominated Sonopiezoelectric Cancer Therapy
Source: Adv Sci (Weinh). 2024 Sep 9;11(42):2405741. doi: 10.1002/advs.202405741 (PMC11558157; doi:10.1002/advs.202405741)
Supplement: Supplementary file 1 — Supporting Information [file ADVS-11-2405741-s001.docx]

**Supporting Information**

**Two-Dimensional Atomically Thin Piezoelectric Nanosheets for Efficient Pyroptosis-Dominated Sonopiezoelectric Cancer Therapy**

*Ruxi Deng, Chunrong Ren, Xinran Song, Wuyang Wei, Hai Wang, Quanyu Nie, Ying Liu, Pan Li, Li Ding, Meiqi Chang*, Yu Chen*, Yang Zhou**

R. Deng, W. Wei, H. Wang, Q. Nie, Y. Liu, Prof. Y. Zhou

Department of Ultrasound, The Third People's Hospital of Chengdu, Affiliated Hospital of Southwest Jiaotong University, Chengdu 610031, Sichuan, P. R. China.

Email: zhouyang@swjtu.edu.cn.

X. Song, Prof. Y. Chen

Materdicine Lab, School of Life Sciences, Shanghai University, Shanghai 200444, P. R. China.

Email: chenyuedu@shu.edu.cn.

Prof. Y. Chen

Shanghai Institute of Materdicine, Shanghai 200051, P. R. China.

A/Prof. M. Chang

Laboratory Center, Shanghai Municipal Hospital of Traditional Chinese Medicine, Shanghai University of Traditional Chinese Medicine, Shanghai 200071, P. R. China.

Email: changmeiqi@vip.sina.com.

Prof. C. Ren

Department of Gastroenterology, The Third People's Hospital of Chengdu, Affiliated Hospital of Southwest Jiaotong University, Chengdu 610031, Sichuan, P. R. China.

Prof. P. Li

Department of Ultrasound, Chongqing Key Laboratory of Ultrasound Molecular Imaging, the Second Affiliated Hospital of Chongqing Medical University, Chongqing 400010, P. R. China.

Prof. L. Ding

Department of Medical Ultrasound, National Clinical Research Center of Interventional Medicine, Shanghai Tenth People's Hospital, Tongji University Cancer Center, School of Medicine, Tongji University, Shanghai 200072, P. R. China.

**Experimental section**

**Chemicals**

Bi(NO_3_)_3_·5H_2_O, alginate hydrogel (ALG), rhodamine B (RhB), polyvinylpyrrolidone(PVP), 2’,7’-dichlorofluorescein diacetate (DCFH-DA), calcein acetoxymethyl ester (Calcein-AM) and propidium iodide (PI) were purchased from Sigma-Aldrich. 3,3′,5,5′-Tetramethylbenzidine dihydrochloride hydrate (TMB), Calcium chloride anhydrous (CaCl_2_), hydrogen peroxide (H_2_O_2_), were obtained from Aladdin. Ethylene glycol (EG, 99%), 5,5-dimethyl-1-pyrroline n-oxide (DMPO), 2,2,6,6-Tetramethyl-4-piperidone hydrochloride (TEMP), and methylene blue trihydrate (MB), 1,3-diphenylisobenzofuran (DPBF) were obtained from Sinopharm Chemical Reagent Co., Ltd, China. PBS, DMEM medium, penicillin/streptomycin, and fetal bovine serum (FBS) were bought from Gibco Life Technologies Co., Ltd (New York, US). 5,5’,6,6’-tetrachloro-1,1’-3,3’-tetraethyl-benzimidazolylcarbocyanine iodide (JC-1) was acquired from Beyotime Biotechnology Co., Ltd (Shanghai, China).

**Preparation of atomically thin Bi_2_O_2_(OH)(NO_3_) nanosheets (AT-BON NSs)**

AT-BON nanosheets were synthesized by a simple top-to-bottom peeling method. First, 6 mmol of Bi(NO_3_)_3_·5H_2_O was dispersed in 60 mL of deionized water and ultrasonicated for 20 min. The obtained solution was then transferred to PTFE-lined stainless autoclave and heated in an oven at 150 °C for 12 h. The solution was separated by centrifugation and washed five times with deionized water. Finally, the powdered product was dried at 60 °C and recorded as BON. Next, the aqueous dispersion of the BON samples was sonicated in air for 6 h. The treated suspension was then centrifuged at 8000 rpm for 1 min. The supernatant was centrifuged at 11,000 rpm for 5 min, and the products were abbreviated as AT-BON NSs.

**Preparation of AT-BON@P NSs**

10 mg of AT-BON powder were dissolved in 10 ml of deionized water, and then 100 mg of PVP was added and stirred for 24 h. Finally, the final products were repeatedly washed through water and ethanol.

**Preparation of AT-BON-G**

AT-BON solid (8 mg) was dispersed in ALG solution (10 mg/mL, 0.9 mL) and kept magnetically stirring for 2 h at room temperature. Then CaCl_2_ solution (10 mg/mL, 0.1 mL) was dropwise added into mixture to fabricate AT-BON-G.

**Characterization**

The morphology of AT-BON was observed by a transmission electron microscope (TEM, JEOL JEM-F200, Japan) and a high-resolution transmission electron microscopy (HRTEM). X-ray photoelectron spectroscopy (XPS, Thermo Scientific K-Alpha, USA) was conducted to study the elemental composition of different NPs. Their crystal phase was obtained using an X-ray diffraction (XRD, Rigaku SmartLab SE, Japan) system with a Co Kα radiation source scanning between 10 and 80 (2θ) degrees. The UV-vis absorption spectrum was obtained on a UV-vis-NIR spectrometer (UV-3600, Shimadzu, Japan). A piezoresponse force microscopy (PFM, Bruker Dimension Icon, German) was used to measure the piezoelectric and ferroelectric properties. Raman spectrum was obtained on LabRam HR Evolution with a laser wavelength of 532 nm. The fluorescence spectrum was detected with a Jobin Yvon FluoroMax-4 fluorescence spectrophotometer equipped with a 150 W xenon lamp as the excitation source.

**ESR measurement**

ESR experiments were performed with the assistance of the trapping agent DMPO and TEMP. 200 μL AT-BON (5 mg mL^-1^) were mixed with 10 μL DMPO or TEMP under US irradiation (1.2 W cm^-2^, 50% duty cycle, 5 min), respectively. Then •OH (DMPO-H_2_O system), ·O_2_^-^ (DMPO-methanol system) and ^1^O_2_ (TEMP-H_2_O system) with the characteristic signals were detected by an electron paramagnetic resonance spectrometer.

**MB degradation**

The •OH generation during the ultrasonic process was detected by the degradation of MB. 20 mg of BON/AT-BON NSs were dispersed in 5 mL MB aqueous solution (5 mg/L). Before imposing US irradiation, the mixture was stirred in dark for 30 min to establish the adsorption-desorption equilibrium. To investigate the degradation efficacy of AT-BON NSs on MB under US irradiation (1.0 MHz, 1.2 W/cm^2^, 50% duty cycle), the mixture was centrifuged and the supernatant was analyzed by UV-vis absorption spectroscopy.

**DPBF fading test**

The generation of ^1^O_2_ via the sonodynamic process was measured using DPBF as a specific probe. 60 µL DPBF (1 mg/mL in ethanol) was mixed with 3 mL AT-BON NSs aqueous suspension (100 µg/mL). Then, the mixture was exposed to US (1.0 MHz, 1.2 W/cm^2^, 50% duty cycle) for fixed time intervals in the dark. The change of DPBF concentration was recorded and calculated by the absorption intensity at 416 nm via UV-vis absorption spectra.

**Cell culture**

4T1 (mouse breast cancer cells) and Hepa1-6 (mouse liver cancer cells) were purchased from the Cell Bank of Shanghai Institute of Biochemistry and Cell Biology, Chinese Academy of Sciences. Cells were cultured in DMEM medium with 10% fetal bovine serum and 1% penicillin/streptomycin at 37 °C in a humidified atmosphere with 5% CO_2_.

**Bio-TEM observation**

4T1 cells were seeded in 6-well plates and cultured for 24 h up to 70-80% confluence. Cells were treated with AT-BON@P NSs and incubated for 6 h. Subsequently, the treated cells were collected, centrifuged, and resuspended in 2.5% glutaraldehyde fixative overnight. After fixation in osmium tetroxide solution for 1 h at room temperature, the cells were then dehydrated with gradient ethanol solutions and embedded in resin. The prepared sections were imaged by TEM.

**Cellular uptake**

Hepa1-6 cells were seeded in 6-well plates and cultured for 24 h up to 70-80% confluence. Then the cell culture medium was replaced with fresh culture medium containing rhodamine B-labeled AT-BON@P NSs (100 µg mL^-1^) for 0, 1, 2, and 4 h, respectively. The treated Hepa1-6 cells were washed twice with PBS for fluorescence microscope observation.

***In vitro* cytotoxicity assay**

4T1/Hepa1-6 cells were seeded into 96-well plates (cell density = 10^4^ cells per disk) and cultured for 24 h. Then, the cell culture medium was refreshed with fresh culture medium containing AT-BON@P with different concentrations, the US-related groups were exposed to the US irradiation (1.0 W cm^-2^, 50% duty cycle, 3 min). Finally, CCK-8 assay was conducted.

**Detection of intracellular ROS production**

4T1 cells were seeded in a confocal glass bottom dish (cell density = 10^5^ cells per disk) and cultured for 24 h. Hepa1-6 cells were seeded and cultured for 24 h. Then, 4T1/Hepa1-6 cells were treated with the following conditions: 1. Control; 2. US (1.0 W cm^-2^, 50% duty cycle, 1 min); 3. AT-BON@P (100 µg mL^-1^); 4. AT-BON@P (100 µg mL^-1^) + US. After co-incubation at 37 °C for 4 h, the US-related groups were exposed to the US irradiation, and then DCFH-DA (10 µM) was added followed by an additional 40 min incubation in the dark. 4T1 and Hepa1-6 cells were washed twice with PBS after different treatments for CLSM and fluorescence microscope observation, respectively.

**Detection of live/dead cells**

4T1/Hepa1-6 cells were seeded in 6-well plates and cultured for 24 h up to 70-80% confluence. Then, 4T1/Hepa1-6 cells were treated with the following conditions: 1. Control; 2. US (1.0 W cm^-2^, 50% duty cycle, 3 min); 3. AT-BON@P (100 µg mL^-1^); 4. AT-BON@P (100 µg mL^-1^) + US. After co-incubation at 37 °C for 8 h, the cells were co-incubated with Calcein AM and PI for 30 min. Finally, 4T1/Hepa1-6 cells were washed repeatedly with PBS and then imaged using a fluorescent microscope.

**Cell apoptosis analysis**

4T1/Hepa1-6 cells were seeded in 6-well plates and cultured for 24 h up to 70-80% confluence. 4T1 cells were treated with the following conditions: 1. Control; 2. US (1.0 W cm^-2^, 50% duty cycle, 3 min); 3. AT-BON@P (100 µg mL^-1^); 4. AT-BON@P (100 µg mL^-1^) + US. Then, 4T1/Hepa1-6 cells were double stained with the Annexin V-FITC Apoptosis Detection Kit (Beyotime-C1062S, Shanghai, China) followed by the flow cytometric analysis.

**Mitochondrial membrane potential disruption assays**

4T1/Hepa1-6 cells (2 mL, 5×10^4^ cells mL^−1^) were incubated into CLSM-exclusive culture disks at 37 °C with 5% CO_2_ for 24 h. Subsequently, 4T1/Hepa1-6 cells were treated with the following conditions: 1. Control; 2. US (1.0 W cm^-2^, 50% duty cycle, 3 min); 3. AT-BON@P (100 µg mL^-1^); 4. AT-BON@P (100 µg mL^-1^) + US. To test mitochondrial membrane potential, all treated cells were washed four times with PBS and then stained with JC-1 (20 µg mL^−1^) for 15 min. The corresponding fluorescence images were characterized by CLSM.

**Detection of intracellular ATP**

The 4T1/Hepa1-6 cells were seeded onto 6-well plates with 400,000 cells per well. They were divided into four groups: 1. Control; 2. US (1.0 W cm^-2^, 50% duty cycle, 3 min); 3. AT-BON@P (100 µg mL^-1^); 4. AT-BON@P (100 µg mL^-1^) + US. After incubation for 24 h, the medium was replaced by fresh medium containing of AT-BON@P NSs. Three hours after US irradiation, the supernatant was collected by lysing cells and then added to the ATP assay kit. ATP content was calculated by the luminescence intensity measured by a BioTek Synergy4 multifunctional plate reader.

**mRNA sequencing and analysis**

4T1 cells were collected using Trizol (Invitrogen) for mRNA sequencing and analysis after treated with control group and AT-BON@P (100 µg mL^-1^) + US (1.0 W cm^-2^, 50% duty cycle, 1 min) group. The mRNA high-throughput sequencing was performed in Personal Biotechnology Co., Ltd. Shanghai, China. The data were analyzed online by using the free platform Personalbio GenesCloud (<https://www.genescloud.cn>). The specific fold-change and P-value thresholds are 2 and 0.05, respectively.

**Lactic dehydrogenase (LDH) assays**

4T1 cells were seeded into 96-well plates and cultured for 24 h. Then, 4T1 cells were treated with the following conditions: 1. Control; 2. US (1.2 W cm^-2^, 50% duty cycle, 3 min); 3. AT-BON@P (100 µg mL^-1^); 4. AT-BON@P (100 µg mL^-1^) + US and incubated. The release of LDH was detected by the Mouse LDH ELISA kit (Yubi (shanghai) Trading Co, LTD).

**Interleukin-1β (IL-1β) secretion**

4T1 cells were seeded into 96-well plates and cultured for 24 h. Then, 4T1 cells were treated with the following conditions: 1. Control; 2. US (1.2 W cm^-2^, 50% duty cycle, 1 min); 3. AT-BON@P (100 µg mL^-1^); 4. AT-BON@P (100 µg mL^-1^) + US and incubated. The release of IL-1β was detected by the Mouse IL-1β ELISA kit (Yubi (shanghai) Trading Co, LTD).

**Western blot analysis**

4T1 cells were seeded in 6-well plates and cultured for 24 h up to 70-80% confluence. Cells were treated with different groups (1. Control; 2. US (1.2 W cm^-2^, 50% duty cycle, 1 min); 3. AT-BON@P (100 µg mL^-1^); 4. AT-BON@P (100 µg mL^-1^) + US and incubated for 8 h, the treated cells were washed with PBS and lysed to collect the protein. The PVDF membranes were blocked with 5% nonfat dry milk at room temperature for 1 h in the decoloring shaker. Then incubated with anti-GSDMD (EPR19828, Abcam), anti-pro caspase-1(EPR16883, Abcam), ASC/TMS (CST, 67824s), anti-Cleaved-Caspase 1 (Ala317), anti-NLRP3 (EPR23094-1, Abcam) overnight at 4 °C. The membranes were washed three times in the decoloring shaker. Then the membranes were incubated with secondary antibodies for 30 min and washed for three times in the decoloring shaker. The electrochemiluminescence reagent was added and reacted for 1-2 min. The membranes were exposed in a darkroom. Finally, the protein expressions were quantified by software Image J.

**Immunofluorescence staining**

4T1 cells were seeded in a confocal glass bottom dish (cell density = 10^5^ cells per disk) and cultured for 24 h. Then, 4T1 cells were treated with the following conditions: 1. Control; 2. US (1.0 W cm^-2^, 50% duty cycle, 3 min); 3. AT-BON@P (100 µg mL^-1^); 4. AT-BON@P (100 µg mL^-1^) + US. After 12 h, 4T1 cells were washed in PBST (0.01 mol/L, containing 0.1-0.2% Triton X-100) for three times, respectively. After blocking with 4% BSA, cells were then incubated with NLRP3 (Novus, NBP2-12446) and ASC/TMS (CST, 67824s) followed by incubation with HRP-labeled secondary antibodies at room temperature. DAPI (Beyotime Biotechnology, C1099, China) counterstaining was used to visualize the nuclei. The immunofluorescent images were acquired via CLSM.

**Animal experiments**

4-week-old Female Balb/c mice were purchased from Shanghai SLAC Laboratory Animal Co., Ltd. The Animal experiment was conducted with the approval of ethics by Ethic Committee of Shanghai University (Approval No. ECSHU-2022-050). The xenograft 4T1/Hepa1-6 tumor models were established by subcutaneously transplanting 4T1/Hepa1-6 cells (1×10^7^, dispersed in 0.1 mL PBS) into the right hind leg of each mouse. When tumor sizes are about ~60 mm^3^, tumor-bearing Balb/c mice were randomly allocated into four groups and injected with intratumoral injections (n = 6): 1. Control; 2. US (1.2 W cm^-2^, 50% duty cycle, 3 min); 3. AT-BON-G (10 mg kg^-1^, 100 μl); 4. AT-BON-G + US (10 mg kg^-1^, 100 μl). On days 0, 2, and 4, US irradiation operation was executed. The body weights and tumor sizes were measured every other day. The tumor volume was calculated using the following formula: tumor volume = length × width^2^/2. The mice were sacrificed after two weeks post-treatment and the tumors were collected. The tumor inhibition rates of each group were calculated using the following equation: tumor inhibition rates = (V_14_, control - V_14_, experiment)/V_14_, control × 100%. Finally, tumors and major organs (heart, liver, spleen, lung, kidney) of different groups were collected and sectioned for H&E staining, Ki-67, TUNEL, cleaved Caspase-1, and NLRP3 assays.

**Supplementary figures**


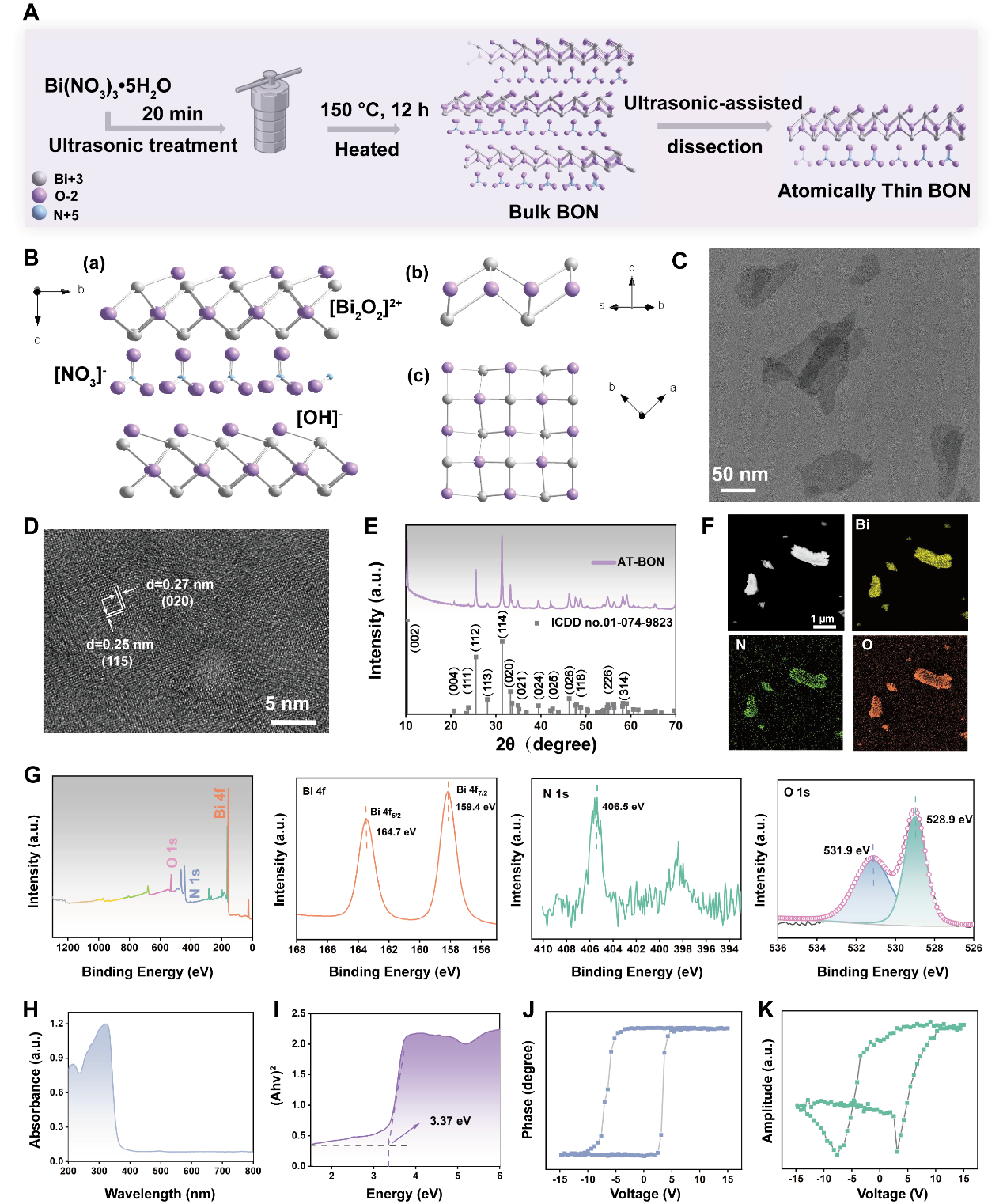


**Figure S1**. Schematic diagram of the synthesis of AT-BON NSs


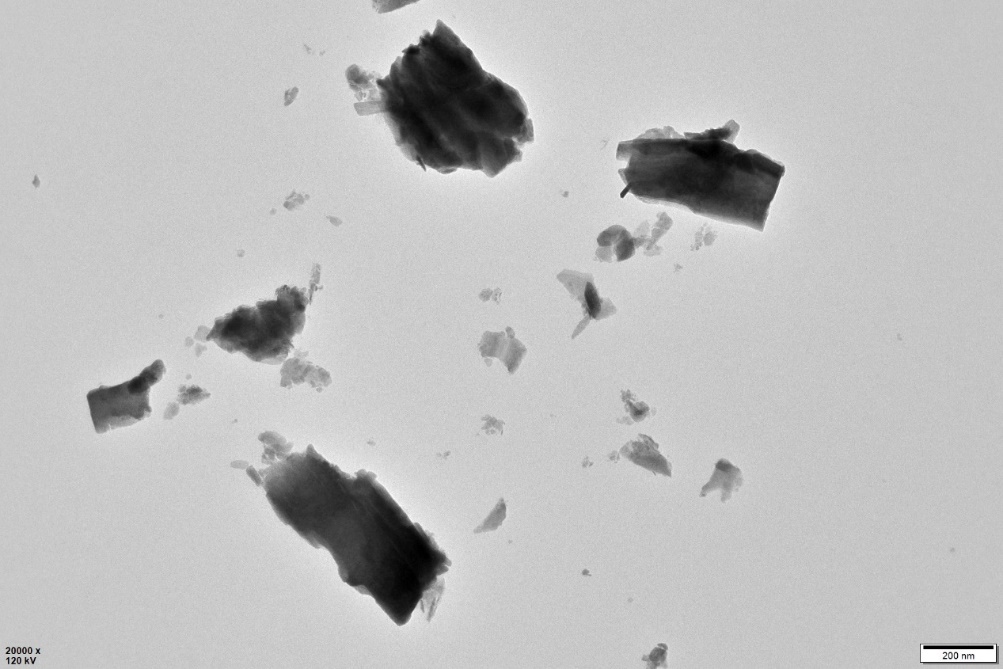


**Figure S2**. TEM image of BON NSs.


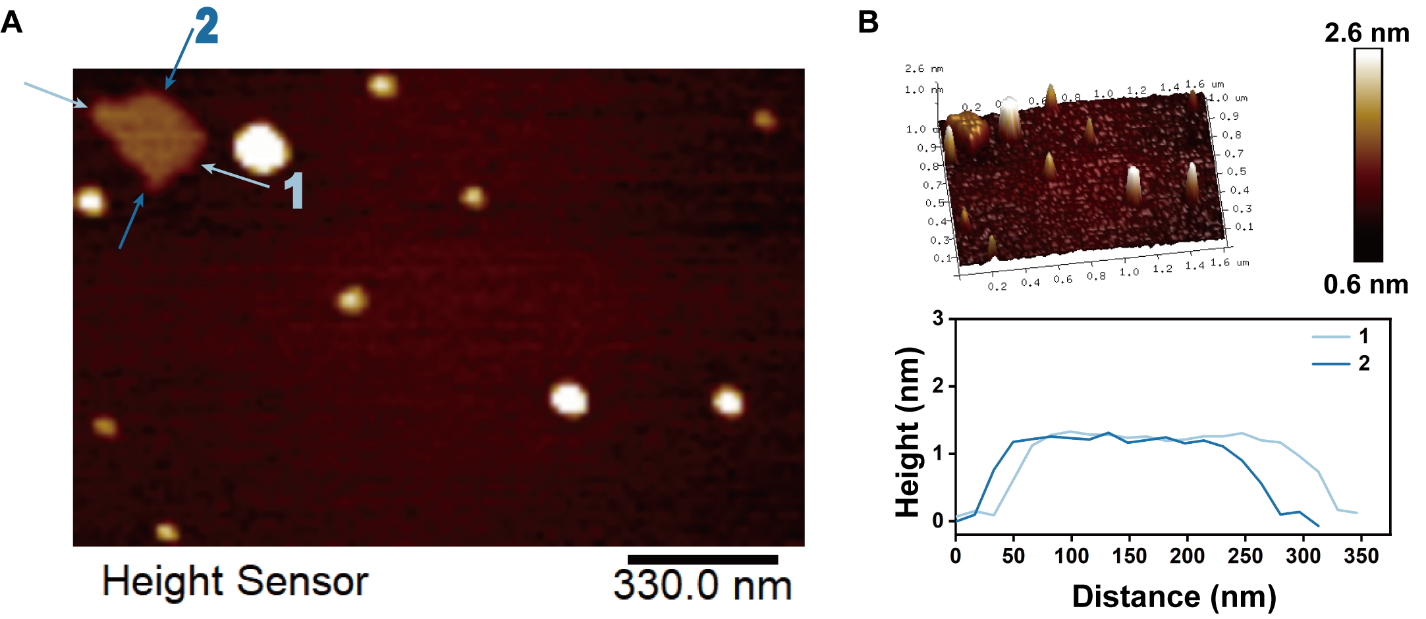


**Figure S3.** A) AFM image and B) 3D images and corresponding height profiles of AT-BON.


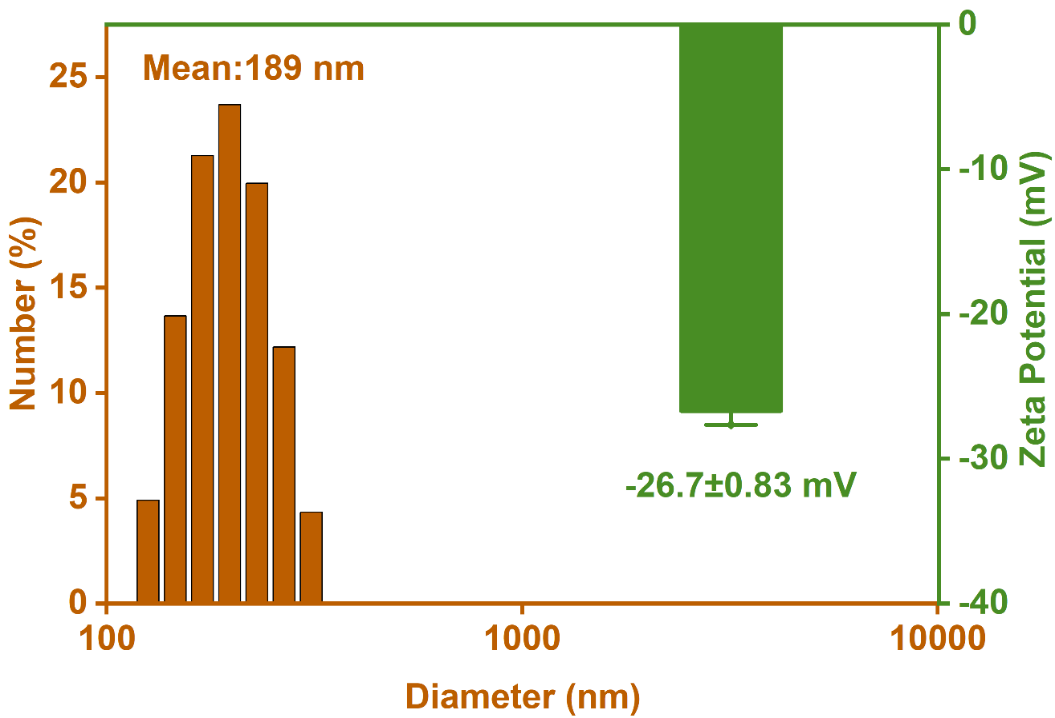


**Figure S4.** Hydrodynamic size distribution and zeta potential of AT-BON.


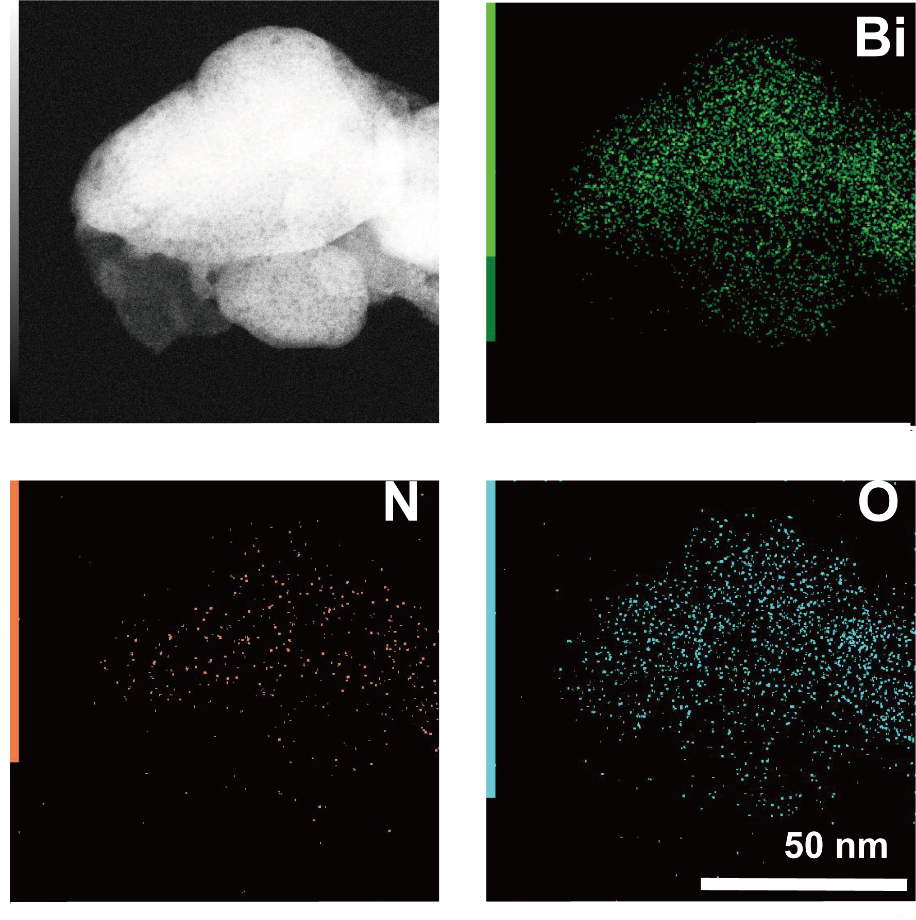


**Figure S5.** Elemental mapping of AT-BON.


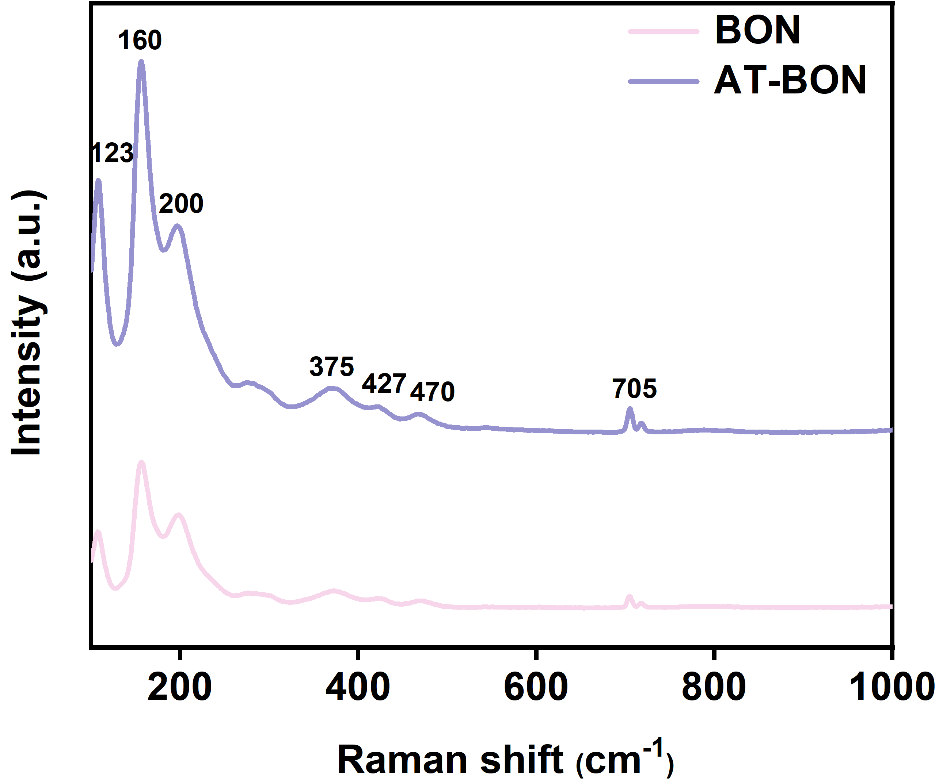


**Figure S6**. Raman spectra of BON and AT-BON.


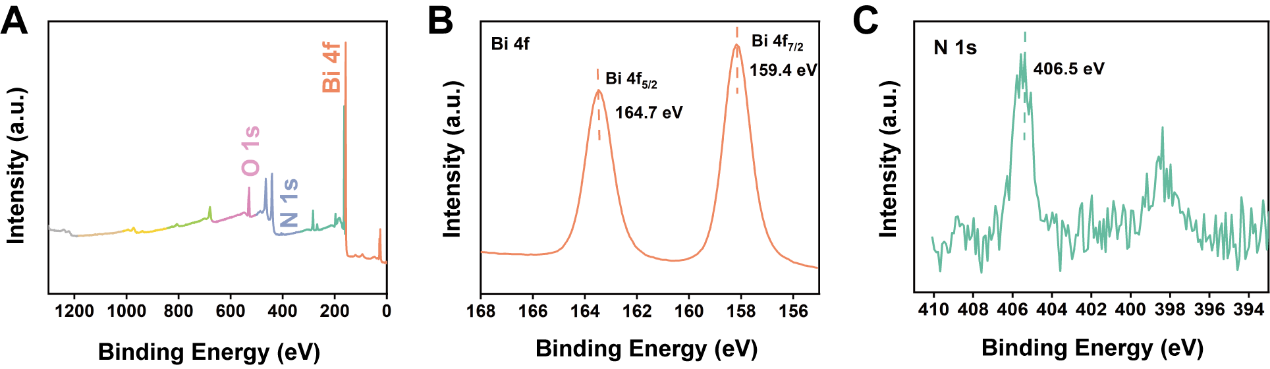


**Figure S7.** XPS spectra of (A) wide-scan, (B) Bi 4f, and (C) N 1s of AT-BON.


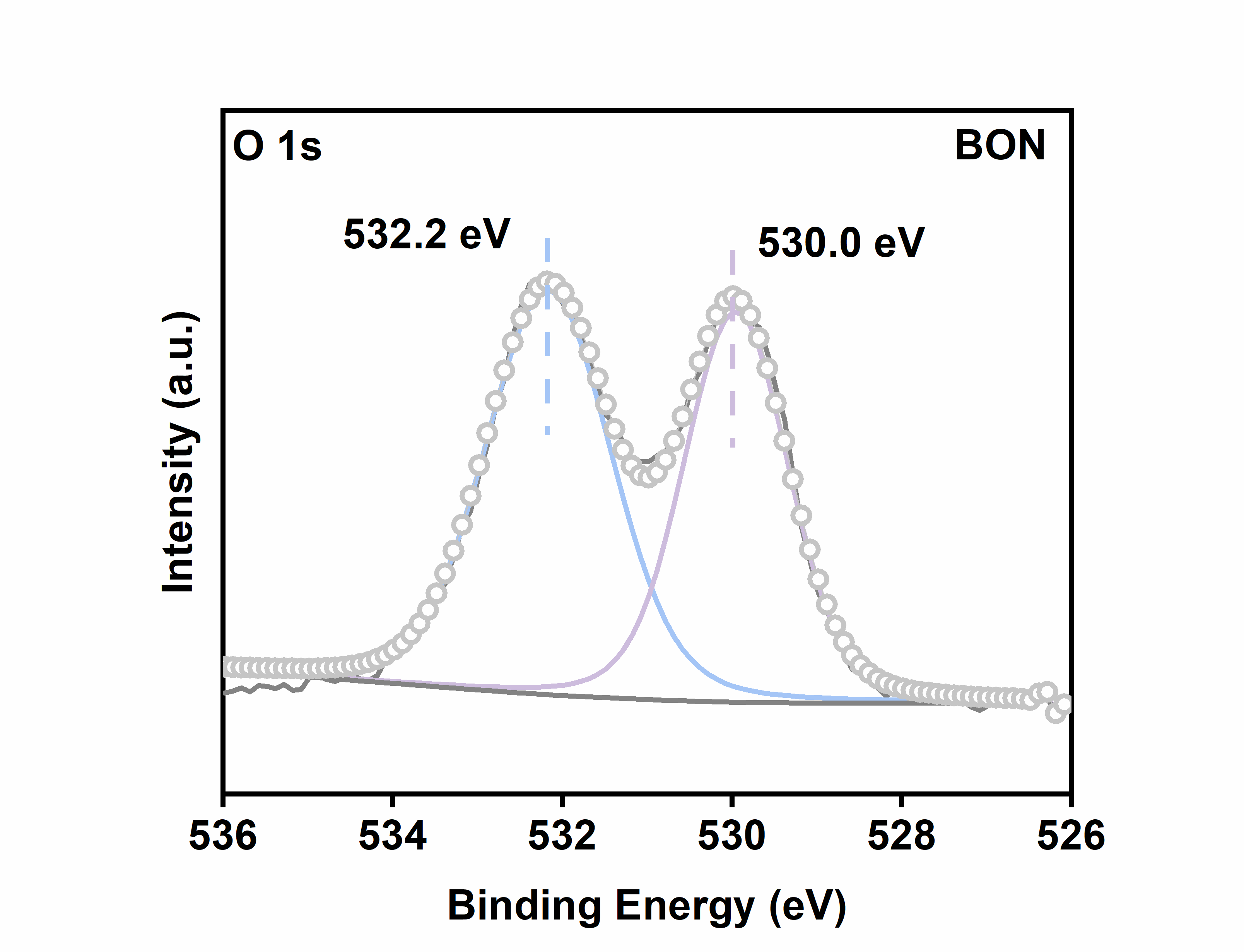


**Figure S8.** XPS spectra of O 1s of BON.


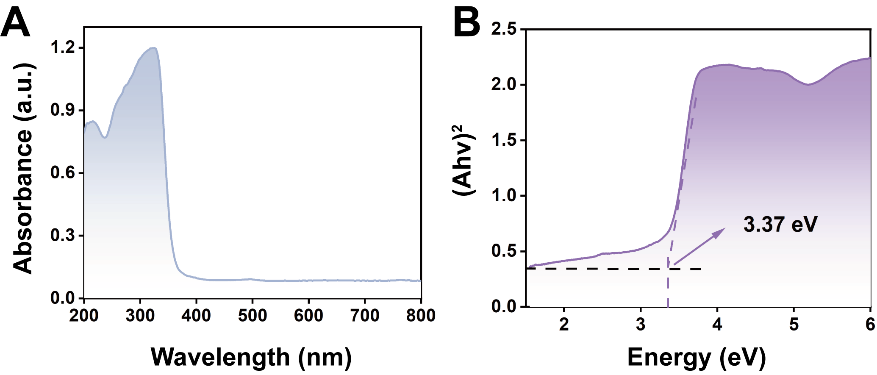


**Figure S9.** (A) UV absorption spectrum and (B) energy bandgap of AT-BON.


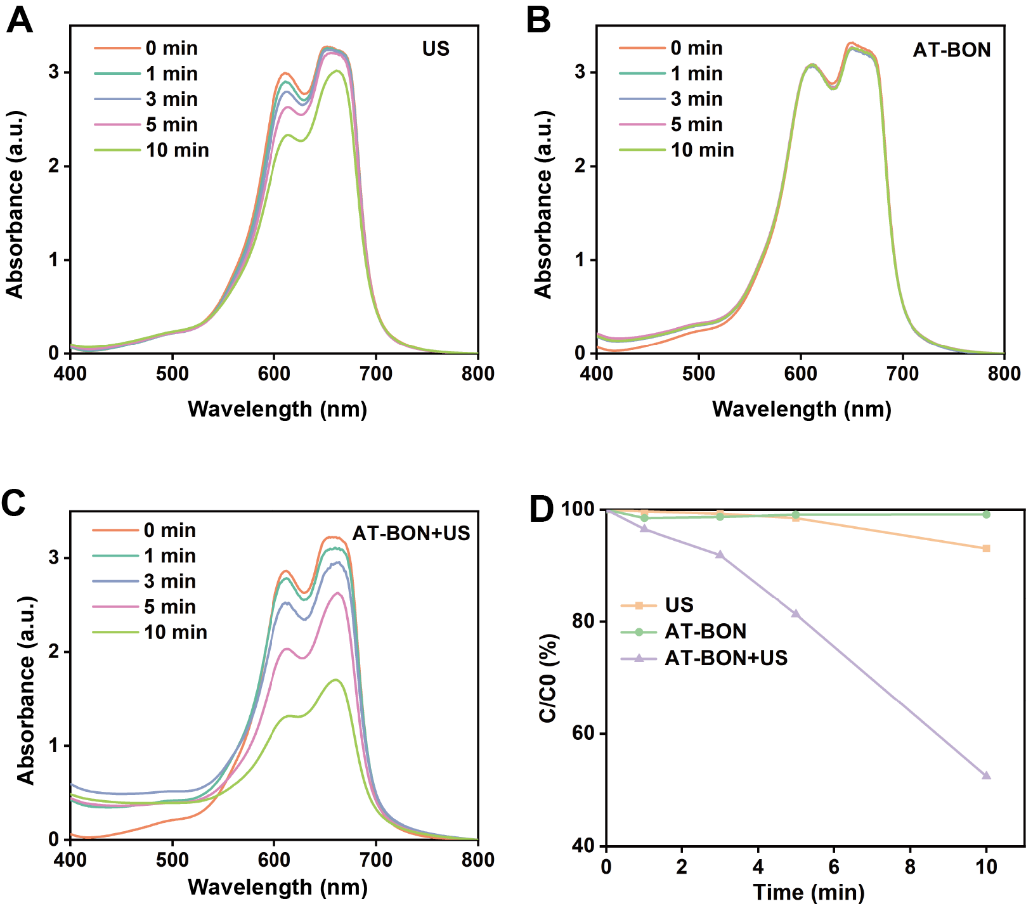


**Figure S10.** Degradation of MB under different treatments. (A) US (1.0 MHz, 1.2 W cm^−2^, 50% duty cycle), (B) AT-BON, (C) AT-BON+US. (D) Comparative curves of MB degradation after different treatments.


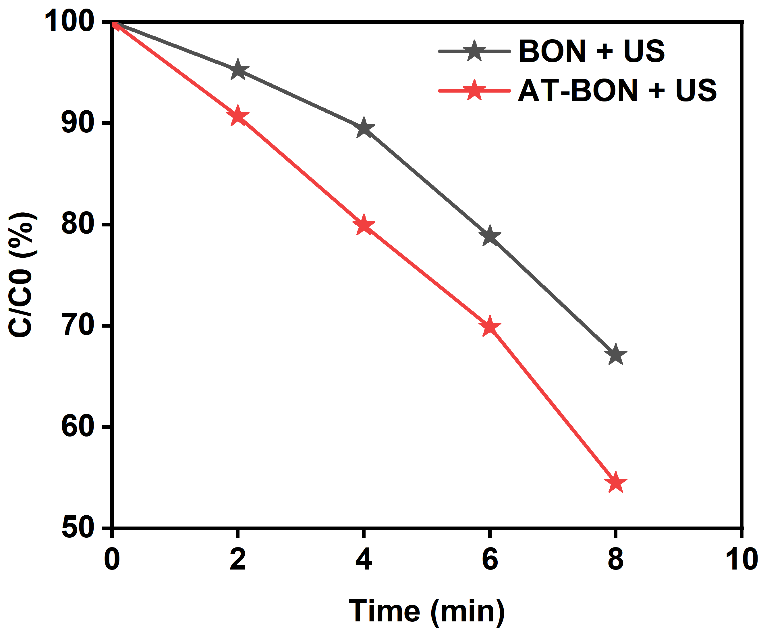


**Figure S11.** Comparative curves of MB degradation after different treatments.
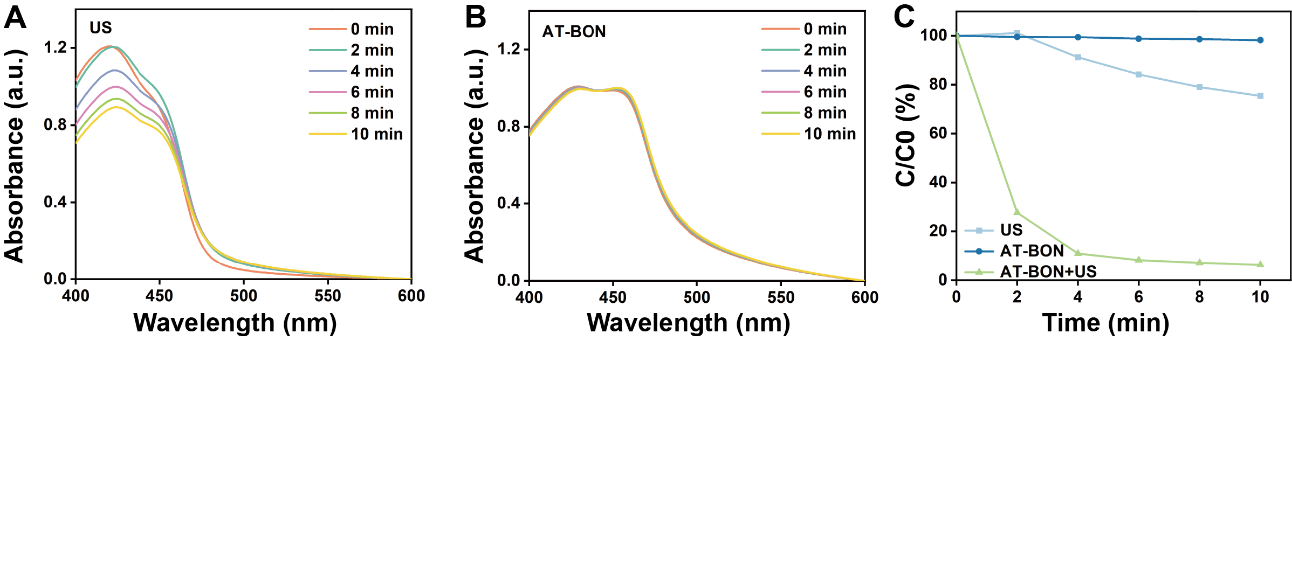


**Figure S12.** Degradation of DPBF under different treatments. (A) US (1.0 MHz, 1.2 W cm^−2^, 50% duty cycle), (B) AT-BON, (C) Comparative curves of DPBF degradation after different treatments.


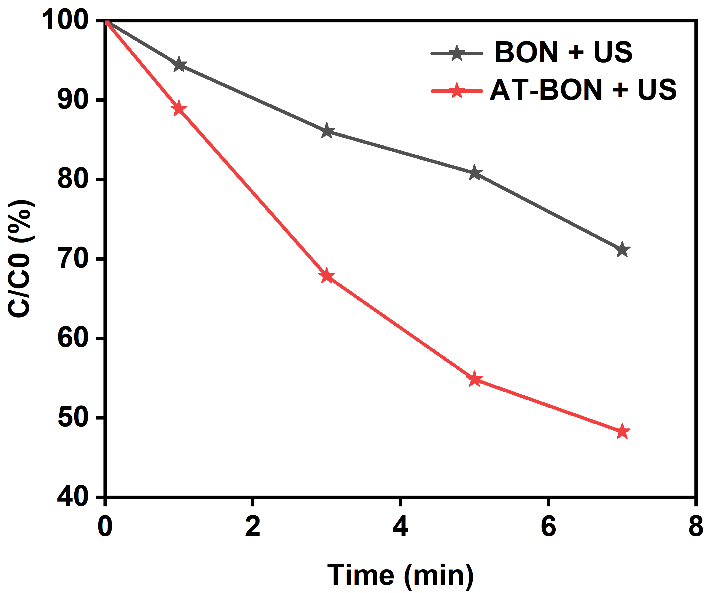


**Figure S13.** Comparative curves of DPBF degradation after different treatments.


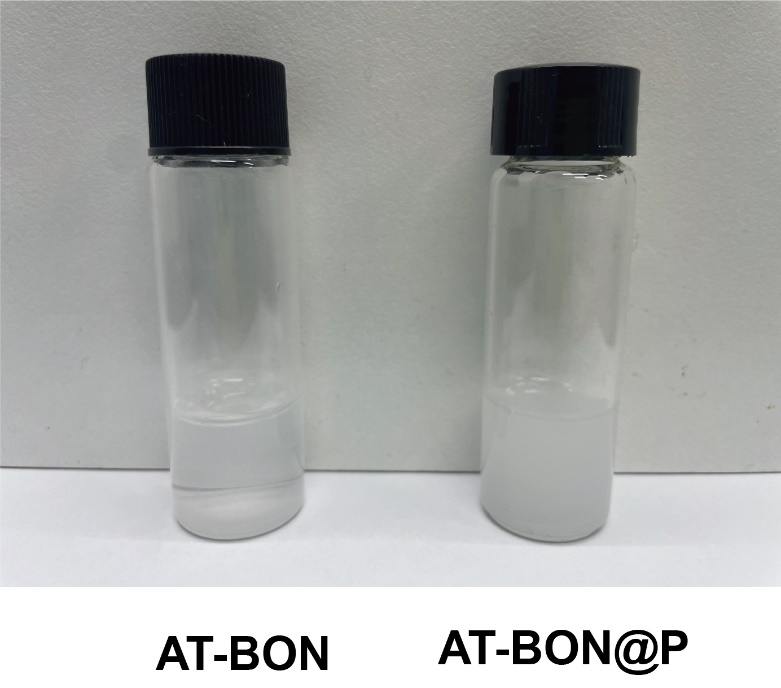


**Figure S14.** Dispersion of AT-BON and AT-BON@P in PBS.


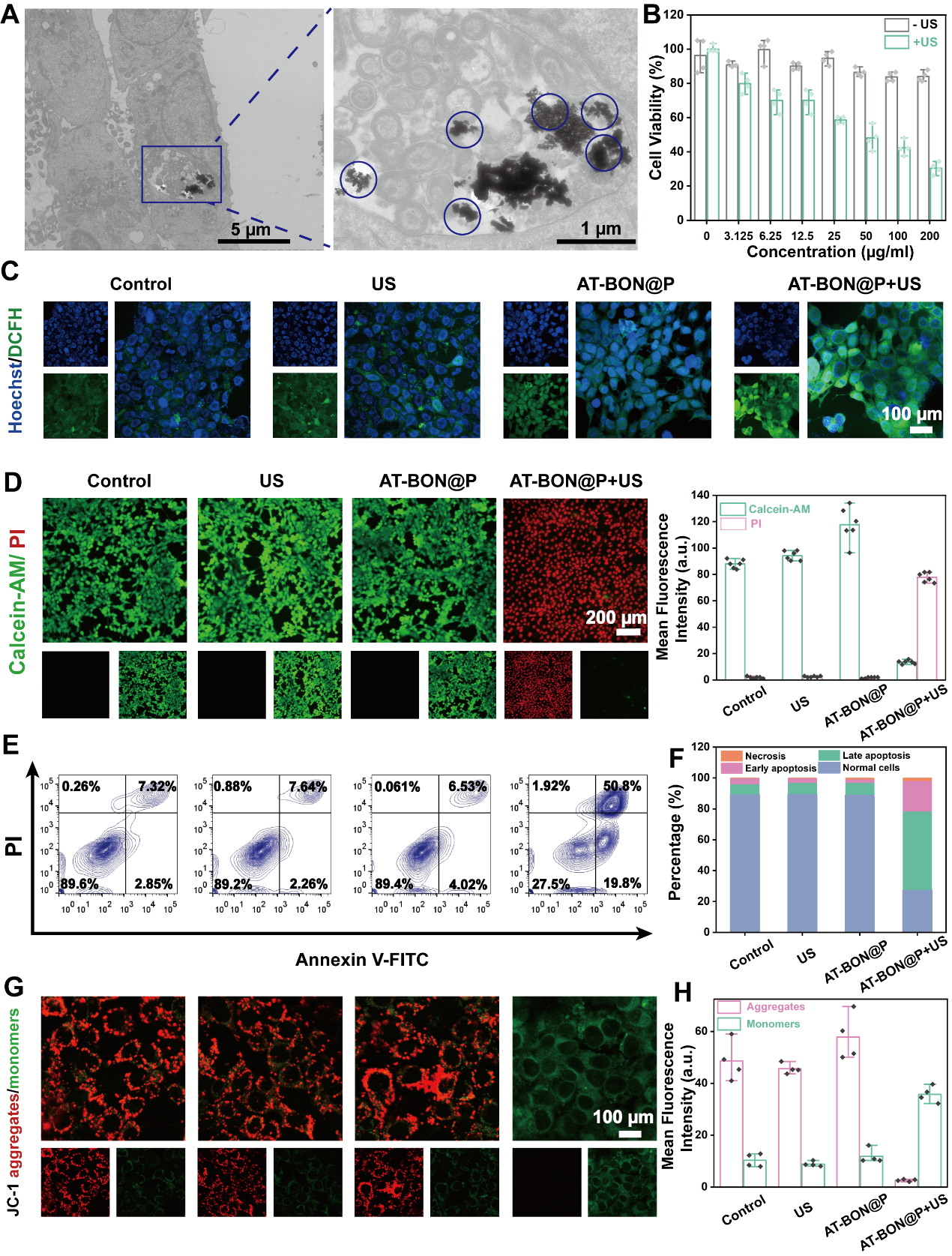


**Figure S15.** Bio-TEM images of 4T1 cells after being incubated with AT-BON@P.

**
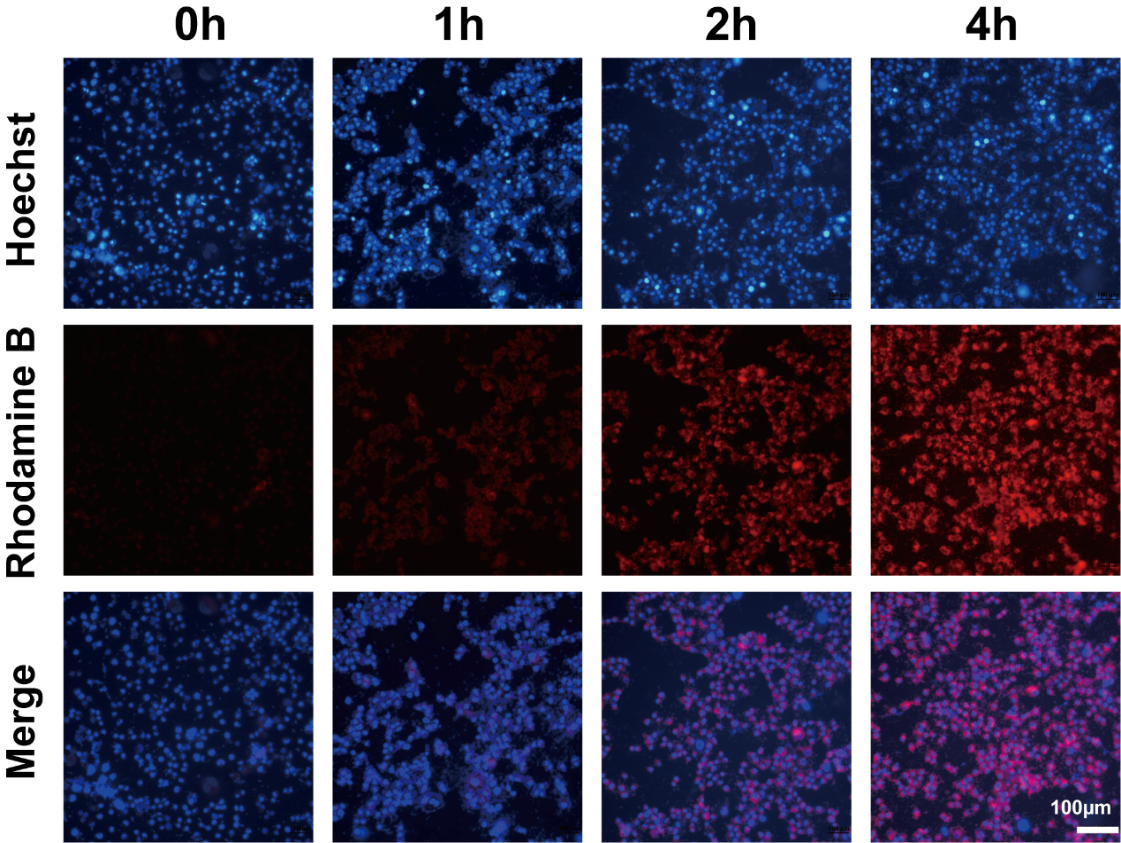
**

**Figure S16.** Hepa1-6 cells incubated with rhodamine B-labelled AT-BON@P (100 µg mL^-1^) at different time intervals.


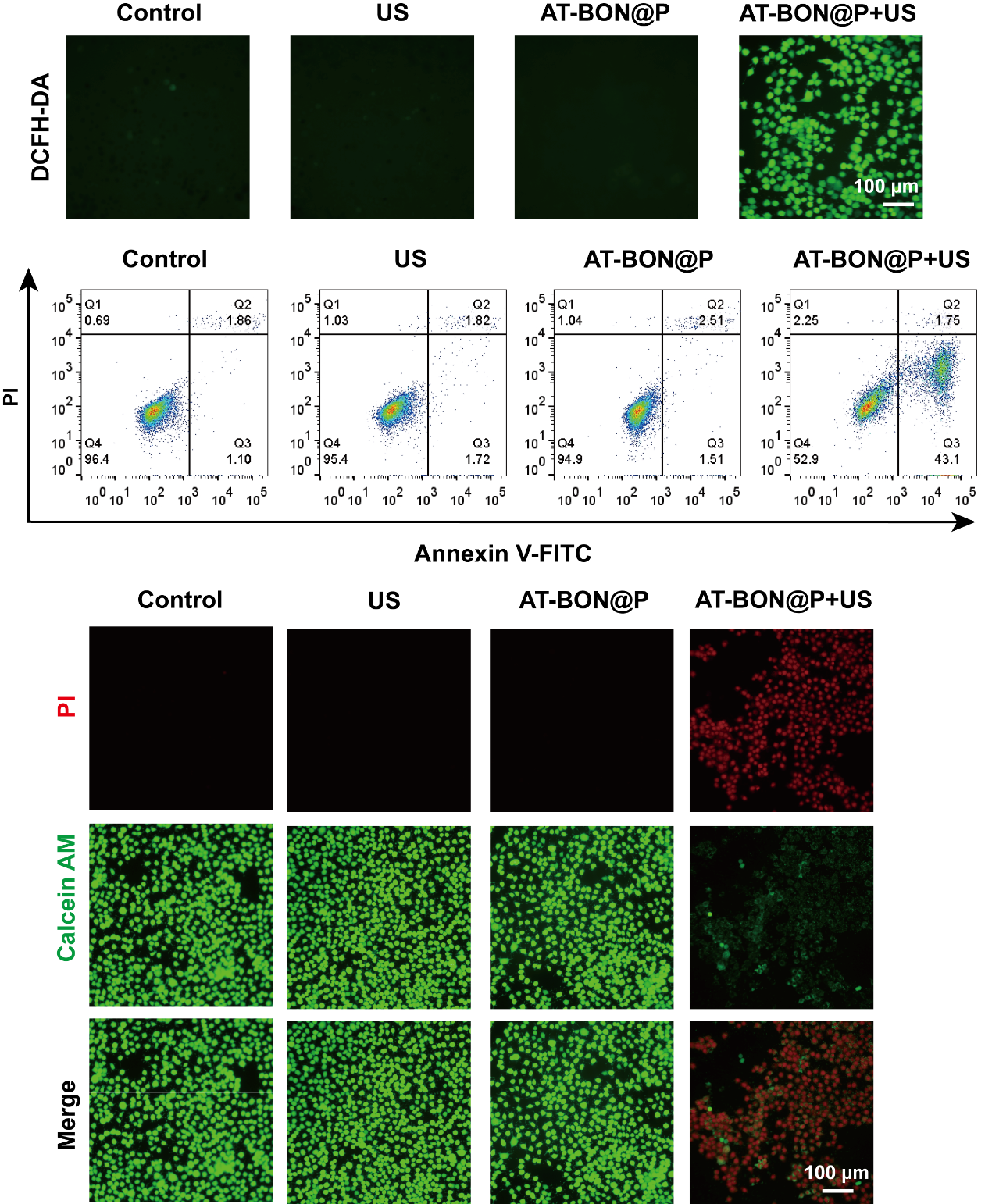


**Figure S17**. Fluorescence images of Hepa1-6 cells stained with DCFH-DA after various treatments.


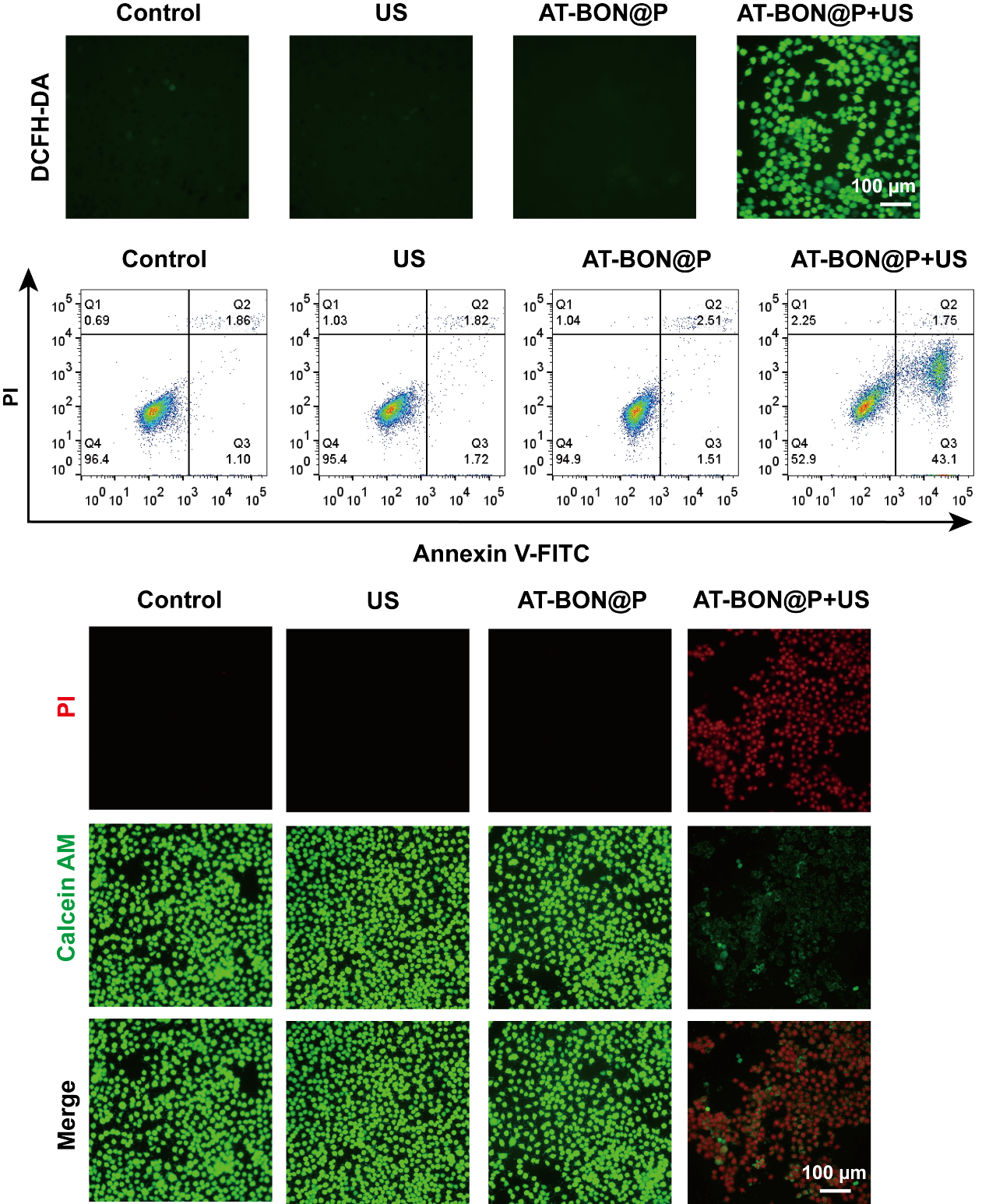


**Figure S18**. Live and dead fluorescence images of Hepa1-6 cells after different treatments, which were stained by Calcein-AM (green) and PI (red).


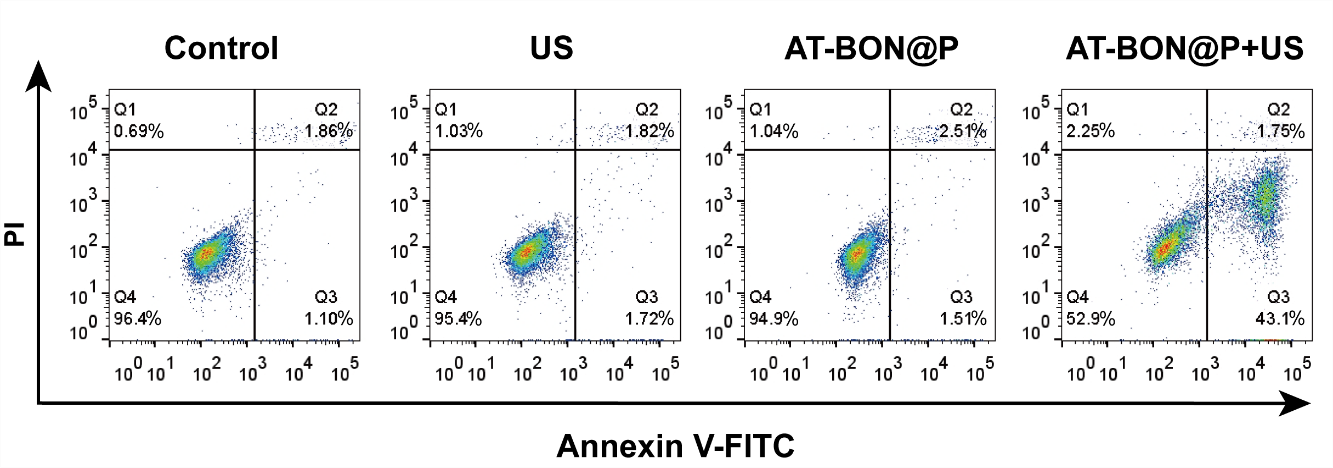


**Figure S19**. Flow cytometric analysis on Hepa1-6 cancer cells co-stained with Annexin V-FITC and PI.


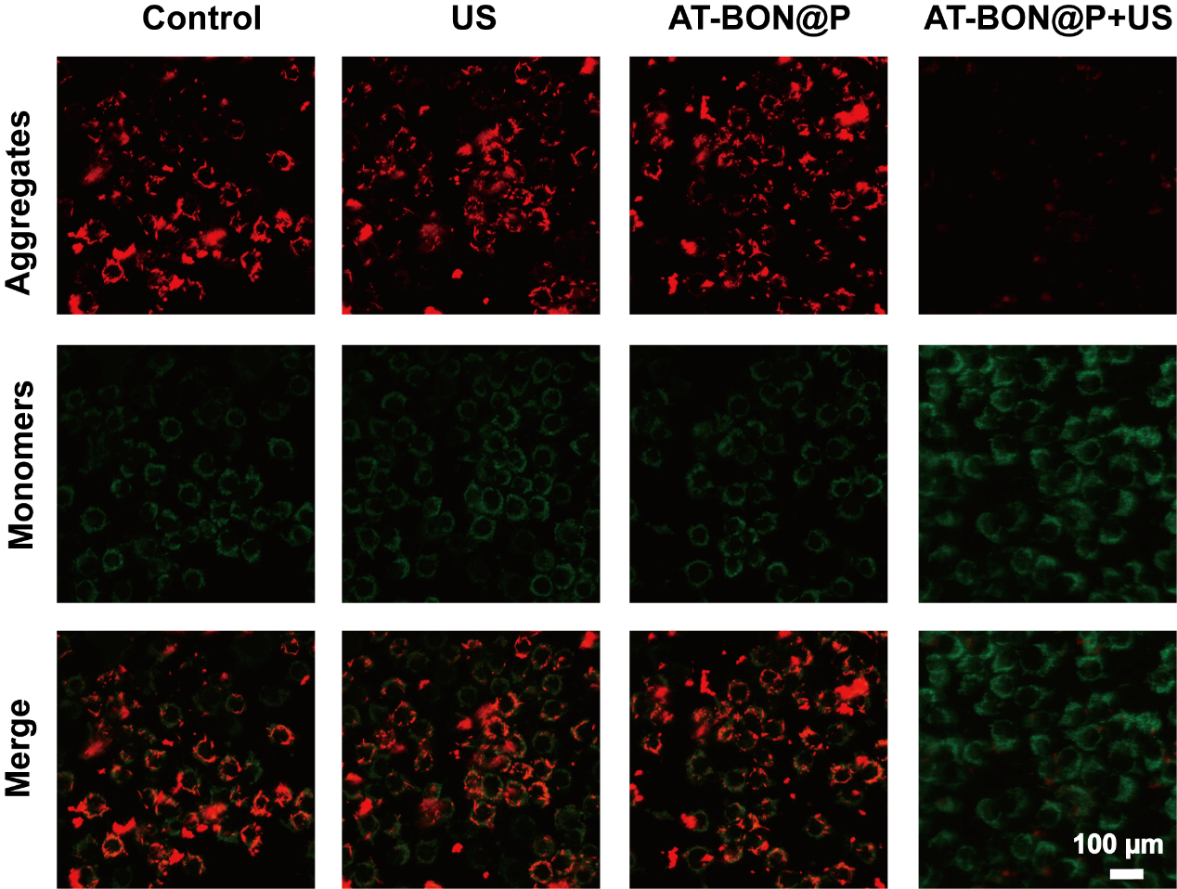


**Figure S20**. JC-1 staining assay of Hepa1-6 cells after different treatments.


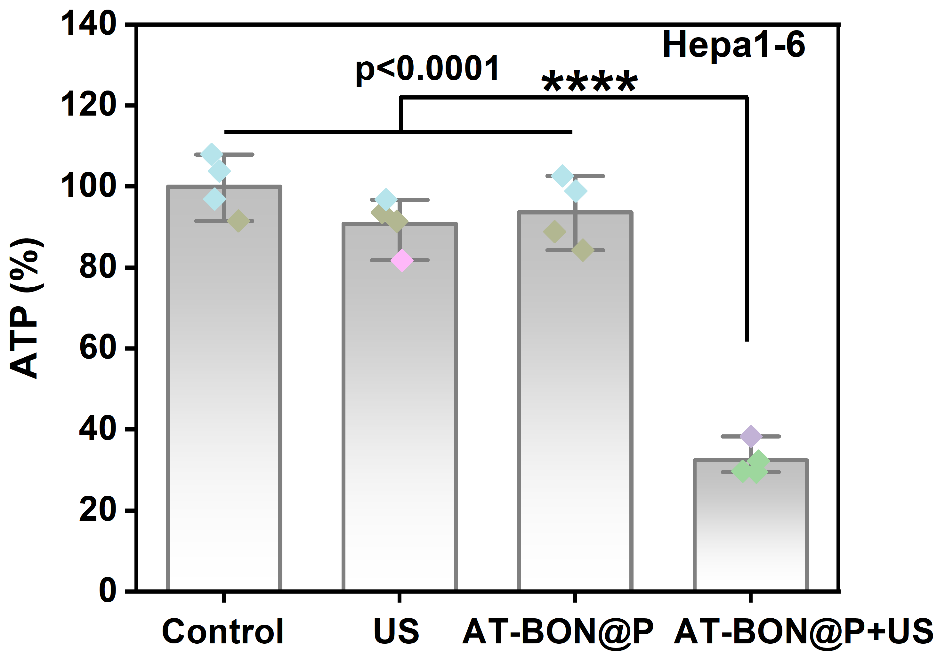


**Figure S21**. The intracellular ATP levels of Hepa1-6 cells after various treatments. Statistical significances were calculated via Student’s t-test. ****p < 0.0001.


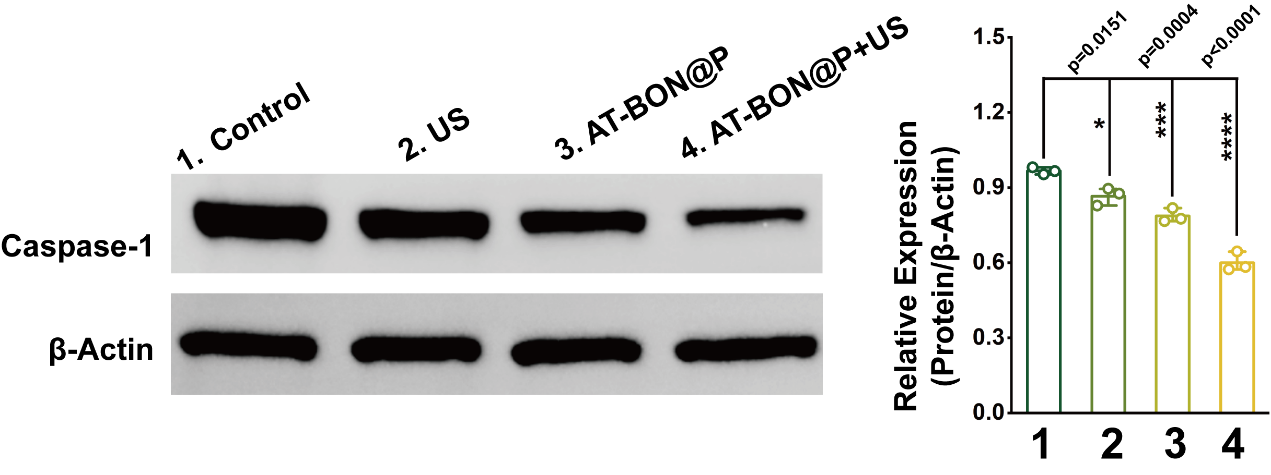


**Figure S22**. Western blot analysis of and corresponding quantitative analysis of Caspase-1 in 4T1 cells after varied treatments. Statistical significances were calculated via Student’s t-test. *p<0.1, ***p < 0.001, ****p<0.0001.


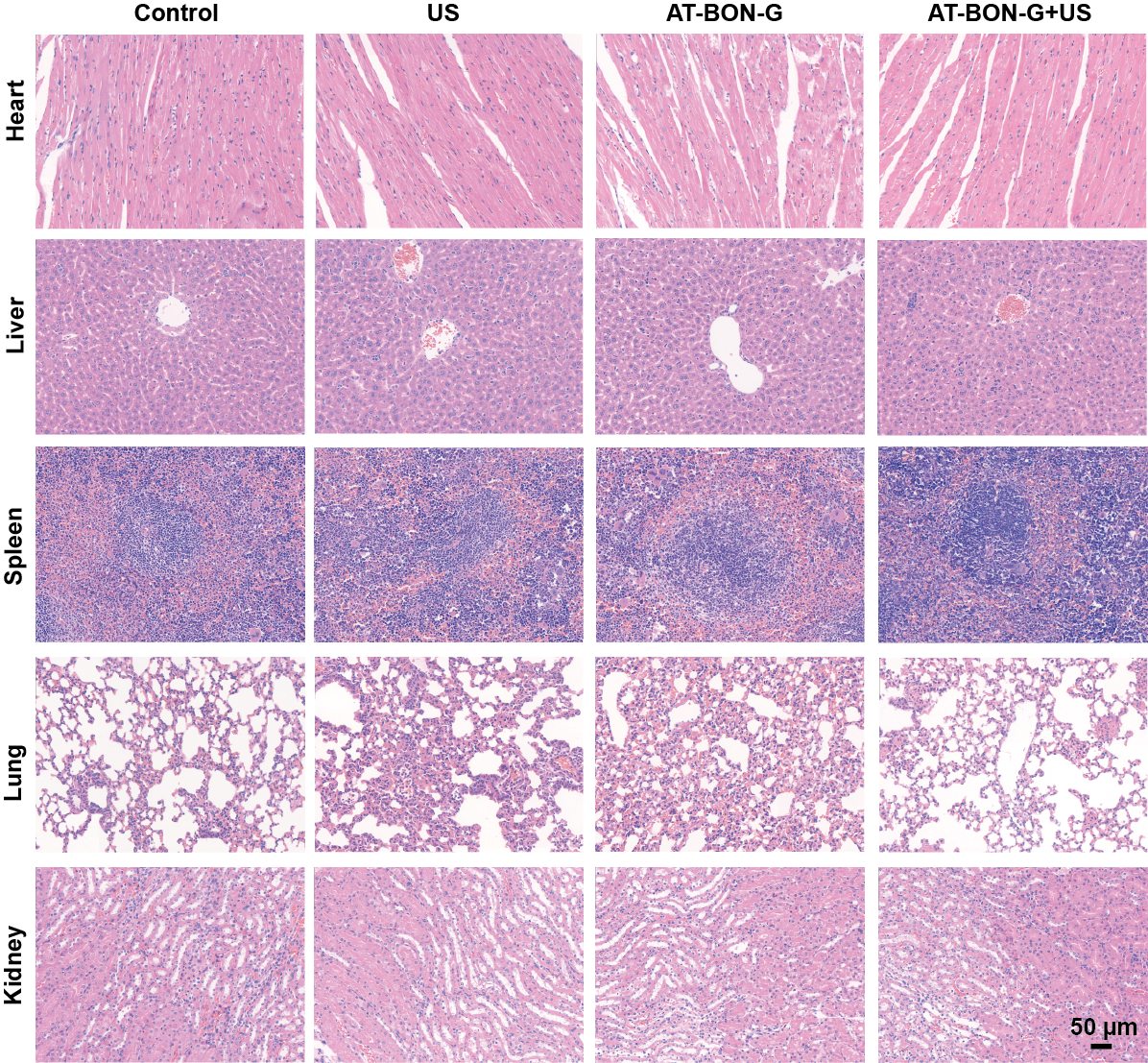


**Figure S23**. Histological examination of major organs (heart, liver, spleen, lung, and kidney) of 4T1 tumor obtained from different treatment groups.


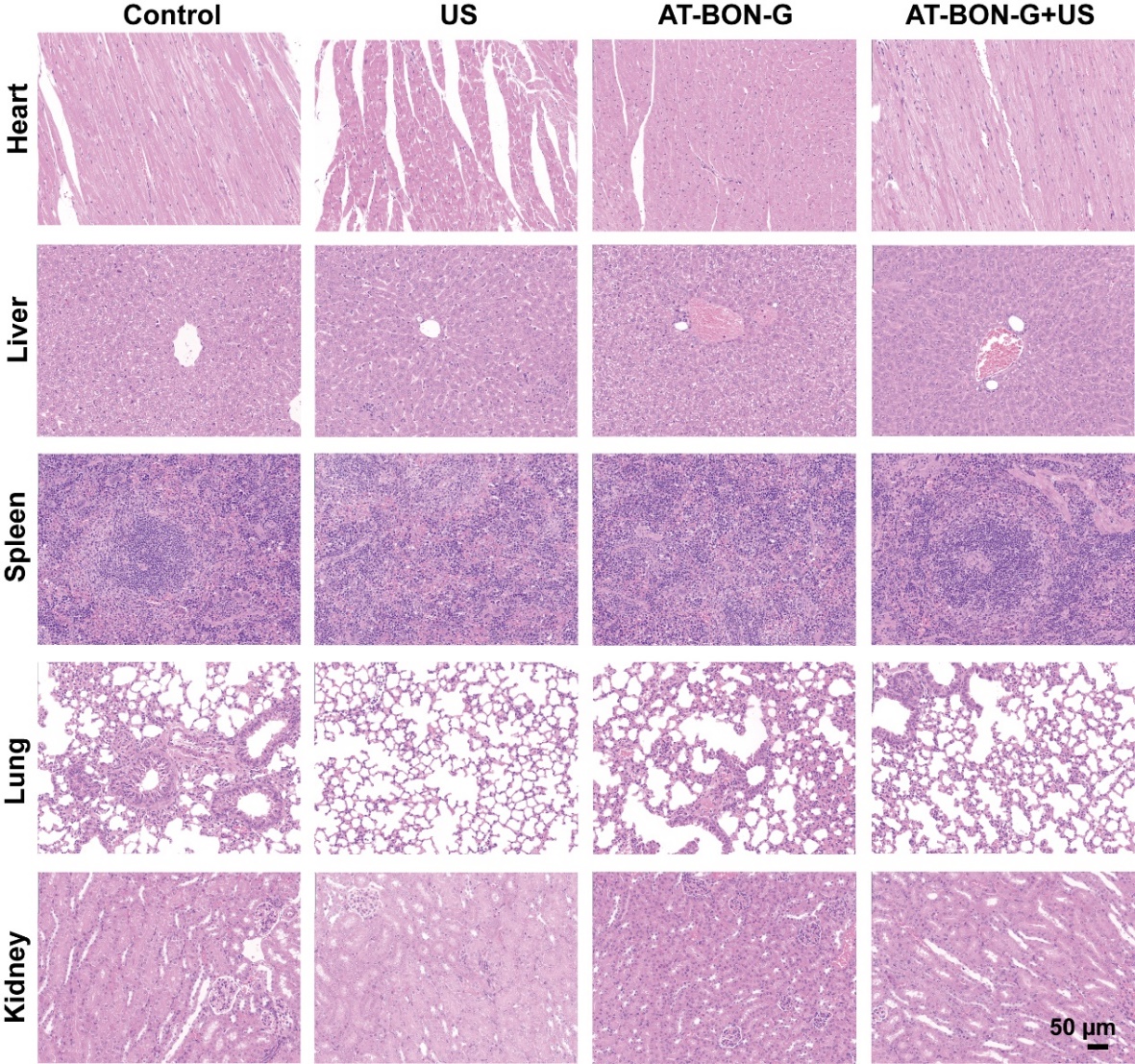


**Figure S24**. Histological examination of major organs (heart, liver, spleen, lung, and kidney) of Hepa1-6 tumor obtained from different treatment groups.


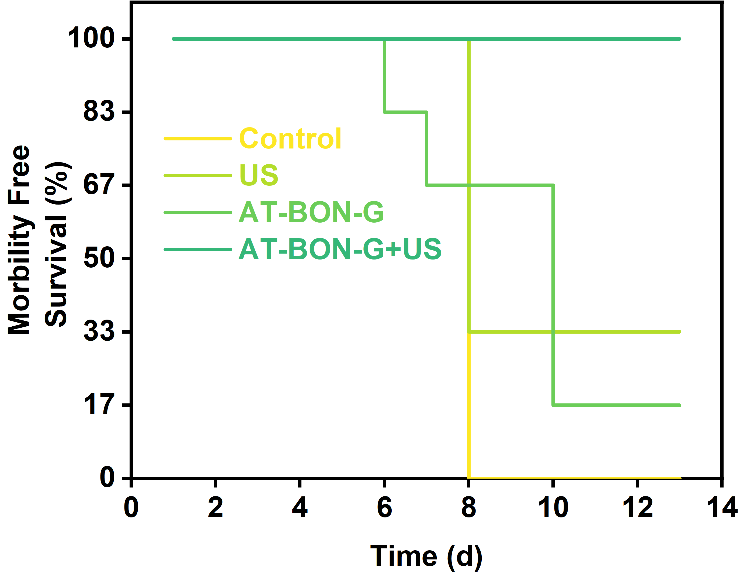


**Figure S25**. The survival rate of Hepa1-6-tumor-bearing mice in different groups.
